# Conditional genome engineering reveals canonical and divergent roles for the Hus1 component of the 9–1–1 complex in the maintenance of the plastic genome of *Leishmania*

**DOI:** 10.1093/nar/gky1017

**Published:** 2018-10-31

**Authors:** Jeziel D Damasceno, Ricardo Obonaga, Gabriel L A Silva, João L Reis-Cunha, Samuel M Duncan, Daniella C Bartholomeu, Jeremy C Mottram, Richard McCulloch, Luiz R O Tosi

**Affiliations:** 1 Department of Cell and Molecular Biology, Ribeirão Preto Medical School, University of São Paulo, Ribeirão Preto 14049-900, SP, Brazil; 2 Laboratório de Genômica de Parasitos, Departamento de Parasitologia, Instituto de Ciências Biológicas, Universidade Federal de Minas Gerais, Belo Horizonte 31270-901, MG, Brasil; 3 Wellcome Centre for Molecular Parasitology, Institute of Infection, Immunity and Inflammation, University of Glasgow, Glasgow G12 8TA, UK; 4 Centre for Immunology and Infection, Department of Biology, University of York, York YO10 5DD, UK

## Abstract

*Leishmania* species are protozoan parasites whose remarkably plastic genome limits the establishment of effective genetic manipulation and leishmaniasis treatment. The strategies used by *Leishmania* to maintain its genome while allowing variability are not fully understood. Here, we used DiCre-mediated conditional gene deletion to show that HUS1, a component of the 9–1–1 (RAD9-RAD1-HUS1) complex, is essential and is required for a G2/M checkpoint. By analyzing genome-wide instability in HUS1 ablated cells, HUS1 is shown to have a conserved role, by which it preserves genome stability and also a divergent role, by which it promotes genome variability. These roles of HUS1 are related to distinct patterns of formation and resolution of single-stranded DNA and γH2A, throughout the cell cycle. Our findings suggest that *Leishmania* 9–1–1 subunits have evolved to co-opt canonical genomic maintenance and genomic variation functions. Hence, this study reveals a pivotal function of HUS1 in balancing genome stability and transmission in *Leishmania*. These findings may be relevant to understanding the evolution of genome maintenance and plasticity in other pathogens and eukaryotes.

## INTRODUCTION

Leishmaniases are a group of life-threatening and disfiguring diseases that are globally widespread (1). These neglected infections are caused by intracellular protozoan parasites of the genus *Leishmania*. Like many other protozoan pathogens, *Leishmania* exploits genome plasticity to survive the inhospitable environments encountered during growth and dissemination. However, the nature and extent of *Leishmania* genome plasticity differs from *Trypanosoma brucei* (2) and *Plasmodium falciparum* (3), parasites whose well known ability to undergo genome rearrangements appears focused on gene families needed for antigenic variation. In contrast, in *Leishmania* species genome plasticity appears to be genome-wide, including gene amplification and chromosome copy number variation, which are hallmarks of genome instability and normally considered detrimental (4,5). Such remarkable genome plasticity can affect the parasite’s gene expression, potentially allowing environmental adaptation (6,7), and has been shown to underlie distinct mechanisms of drug resistance, hampering the establishment of effective antileishmanial chemotherapy (8). Genome plasticity also hinders genetic manipulation of the parasite, making the understanding of its biology even more challenging.

The potential novelties in genome maintenance that underlie the generation and tolerance of *Leishmania* genome variation, and hence the balance between stability and variability, are still poorly understood. RAD51 and MRE11 are key DNA repair proteins that have been shown to play crucial functions in determining the nature and abundance of *Leishmania* amplicons (9–11). Their characterization constitutes an important advance in dissecting the factors required for adaptive amplification and gene rearrangements in *Leishmania*. However, the signaling events that direct these activities, the factors that dictate chromosome ploidy variation, and the dynamics of genome maintenance in this organism are still not well understood. The Rad9–Rad1–Hus1 (9–1–1) complex is a ring-shaped trimeric clamp that is loaded onto DNA in response to replication stress, serving as a scaffold for the association of downstream factors that can lead to cell-cycle arrest until single strand DNA resulting from replication stress is resolved (12,13). In addition, the 9–1–1 complex is involved with DNA damage repair and telomere maintenance (14–16). Thus, disruption of 9–1–1 complex frequently leads to genomic alterations, revealing its key role in safeguarding genome stability in eukaryotes.

We have previously shown that HUS1 is involved in the *Leishmania major* response to genotoxic stress (17,18), but the roles that are critical for the parasite’s survival have not been determined. In this study, we have adapted the DiCre-mediated gene deletion system (19,20) to be used in *L. major* and reveal the essentiality of HUS1. We have advanced our understanding of HUS1 function at the G2/M checkpoint by demonstrating that its absence leads to aberrant mitosis onset in the presence of DNA damage in both unperturbed and replication-stressed cells. Also, genome-wide analysis revealed at least two further, distinct roles of HUS1. Under non-stressed conditions, HUS1 ablation led to increased genomic variability, confirming its role in preventing genome instability. However, in cells exposed to chronic replication stress, HUS1 ablation led to a substantial decrease in variability, revealing an unpredicted and divergent role by which HUS1 contributes to *Leishmania* genome variation. These different effects of HUS1 absence correlated with distinct patterns of DNA damage and cell-cycle progression. We also show that the genome-wide instability dictated by the divergent roles of HUS1 correlates with the peculiar dynamics of the parasite’s DNA replication. Thus, our findings demonstrate the conservation of HUS1 function as a guardian of genome stability and also uncover novel roles in the promotion of genome variation in *Leishmania*, which indicate diverged functions relative to canonical eukaryotic 9–1–1 checkpoint clamp. The implications of these findings for the biology of the parasite are discussed.

## MATERIALS AND METHODS

### Parasite culture

Cell lines were derived from *L. major* LT252 (MHOM/IR/1983/IR) and cultured as promastigotes in M199 medium with 10% heat-inactivated fetal bovine serum at 26°C. DNA fragments were transfected into exponentially growing cells by electroporation with Amaxa Nucleofactor™ II using manufactory pre-set program U-033. After electroporation, transfectants were selected in 96-well plates by limiting dilution with medium containing the appropriate selecting drug. *HUS1*^Flox^ cells were selected with 50 μg/ml nourseothricin, 10 μg/ml blasticidin and 10 μg/ml puromycin. *HUS1*^Flox^AB cells were selected with 50 μg/ml nourseothricin, 10 μg/ml blasticidin, 10 μg/ml puromycin and 8 μg/ml G418.

### DNA constructs

The DiCre (pGL2339 (20)) and *HUS1*^Flox^-expressing constructs were generated by gateway recombination reactions. For the DiCre construct, a BP reaction was performed to introduce the 5′SSU (253 bp) and 3′SSU (955 bp) homology sequences into pDONR P41-Pr and pDONR P2rP3 vectors, respectively. Then, the resulting constructs were used in a LR reaction with the pGL2313 (19) and pDEST vectors to generate the pGL2339 vector. This vector was then digested with *Pac*I and *Pme*I and used for transfection of *∧hus1::NEOSAT/HUS1* cell line, to generate the *∧hus1::NEOSAT/HUS1[SSU DICRE]* cell line.

The same strategy was used to generate the HUS1^Flox^ expressing construct. HUS1 ORF (LmjF.23.0290) was cloned into the *Nde*I restriction site of pGL2314 (19) to generate the pGL2314*HUS1*^Flox^ vector. BP reaction was carried out to introduce the 5′UTR (547 bp) and 3′UTR (486 bp) homology sequences of HUS1 into pDONR P41-Pr and pDONR P2rP3 vectors, respectively. Then, the resulting constructs were used in a LR reaction with the pGL2314*HUS1*^Flox^ and pDEST vectors to generate the pGL2314-5UTR/*HUS1*^Flox^/3UTR vector. This vector was then digested with *Pac*I and *Pme*I and used for transfection of the *∧hus1::NEOSAT/HUS1[SSU DICRE]* cell line to generate the *∧hus1::NEOSAT/ Dhus1::HUS1^Flox^[SSU DICRE]* cell line (referred as the *HUS*1^Flox^ cell line throughout the text; also see Supplementary Figure S1a–c). The pXG1NEO-*HUS1* and pXG1NEO-*RAD9* vectors used in the add-back cell lines were previously described (17). Briefly, *L. major HUS1* and *RAD9* ORFs (LmjF.23.0290 and LmJF.15.0980, respectively) were polymerase chain reaction (PCR) amplified and cloned into the *Xma*I site of pXG1NEO vector (21). The undigested pXG1NEO-*HUS1* and pXG1NEO-*RAD9* vectors were used for transfections of the *HUS*1^Flox^ cell line to generate the *HUS*1^Flox^AB and *HUS*1^Flox^*RAD9* cell lines, respectively.

### DNA extraction

Cells were harvested and total DNA was extracted with DNeasy Blood & Tissue Kit (QIAGEN) following the manufacturer instructions.

### Genome sequencing and bioinformatics analysis

Whole genome sequencing was performed by Glasgow Polyomics (http://www.polyomics.gla.ac.uk/index.html), using a NextSeq™ 500 Illumina platform, generating paired end reads of 100 nt. The quality of each read library was evaluated with FASTQC (http://www.bioinformatics.babraham.ac.uk/projects/fastqc/), and filtered using Trimmomatic. The phred quality filtering threshold was a minimum of 20, using 5 nt sliding window, as well as a minimum read size of 35 nt. Reads were mapped to the *L. major Friedlin* version 26 reference genome (available at Tritrypdb - http://tritrypdb.org/tritrypdb/) using BWA-mem (22). SAMtools v1.18 (23) was used to filter reads with a mapping quality score of 30. Single nucleotide polymorphisms (SNPs) were obtained using the SAMtools function mpileup (24). To minimize identification of false positive events, only those SNPs with a minimum number of 10-mapped reads were considered. Also, in order to reduce the mapping bias of collapsed and repetitive regions, SNPs that presented a coverage that exceeded twice the genome coverage were excluded. The genome localization and specificity of the SNPs were obtained by in house Perl and Bash scripts. All types of SNPs (synonymous and non-synonymous, and also those found in intergenic regions) were considered for the subsequent analysis. Mapped reads were subjected to paired-end mapping analysis component and also to split-read analysis component using DELLY with the default settings (25) for the identification of other genomic structural variants (deletions, insertions, duplications and translocations). The heatmaps and violin plots were generated by Excel and R studio, respectively (www.r-project.org, R Development 2010).

### Antibodies

Generation of affinity purified antibodies anti-LmRad9 (1: 3000), anti-LmHus1 (1: 2000), anti-Rpa1 (1: 1000), anti-γH2A (1: 5000) from rabbit serum, and chicken anti-Rad1 (1: 2000) from eggs, was previously described (17,18,26). Commercial primary antibodies used here were: mouse anti-Myc (1: 5000), anti-EF1α (1: 40 000) and anti-β-Tubulin-clone KMX1 (1: 1000) (Merck Millipore); mouse anti-BrdU (1: 500) (BD Bioscience); rabbit anti-H2A (1: 2000) (Santa Cruz). Commercial secondary antibodies used here were: goat anti-Rabbit IgG Alexa 488, anti-Rabbit IgG Alexa 594, anti-Mouse IgG Alexa 488 and anti-Mouse IgG Alexa 594 (Thermo Fisher).

### Western blot

Sodium dodecyl sulphate-polyacrylamide gel electrophoresis (SDS-PAGE) was used to resolve proteins that were then transferred to Polyvinylidene difluoride (PVDF) membrane. Before probing for specific proteins, membranes were blocked with 10% (w/v) non-fat dry milk in phosphate-buffered saline (PBS). ECL Prime Western Blotting Detection Reagent (GE Life Sciences) was used for band detection as visualized with Hyperfilm ECL (GE Life Sciences). For detection of Rad9, Rad1 and Hus1, western blotting analysis was performed with SuperSignal^®^ Western Blot Enhancer (Thermo Scientific), following manufacturer instructions.

### Immunofluorescence

Cells were washed with PBS, fixed with 4% paraformaldehyde for 15 min, washed with PBS and stored in PBS at 4°C until use. Cells were adhered to glass slides coated with poly-L-lysine and permeabilized with 0.3% Triton X-100 for 20 min. Cells were pre-blocked with 1% bovine serum albumin (BSA) in PBS for 1 h. Cells were incubated with anti-Myc, anti-BrdU or anti-γH2A antibody at 1:500 dilution in PBS for 1 h. Primary antibodies were visualized with the appropriate secondary antibodies conjugated with either Alexa Fluor 488 or Alexa Fluor 594. DNA was stained with Hoechst 33342 and cells were mounted with ProLong^®^ Gold Antifade Reagent (Thermo Fisher).

### Fluorescence-activated cell sorting (FACS)

For DNA content analysis, cells were harvested, washed with PBS and fixed in 30% PBS/70% methanol for at least 16 h at 4°C. Then, cells were washed with PBS and DNA was stained with PBS containing Propidium Iodide (10 μg/ml) supplemented with RNase A (10 μg/ml) for 15 min at 37°C. Flow cytometry data were collected using a BD FACSCanto flow cytometer. Data were analyzed using the FlowJo software. For analysis of both DNA content and ssDNA or γH2A levels, cells were fixed in 30% PBS/70% ethanol for at least 16 h at −20°C. Then, cells were washed with PBS with 1% BSA and incubated with anti-BrdU or anti-γH2A at 1:300 dilution in PBS with 1% BSA for 1 h. Cells were washed with PBS with 1% BSA, blocked with PBS with 3% BSA for 30 min and then incubated with the appropriated Alexa Fluor conjugated secondary antibody in PBS with 1% BSA for 1 h. After washing with PBS with 1% BSA, DNA was stained with PBS containing Propidium Iodide (10 μg/ml) supplemented with RNase A (10 μg/ml) for 15 min at 37°C. Data were collected using a BD FACSCanto flow cytometer. Data were analyzed using the FlowJo software.

### Cell fractionation

Protocol for preparation of soluble and chromatin bound proteins were previously described (17). Briefly, cells were harvested, washed with PBS and suspended in extraction buffer (10 mM Tris–HCl pH 9.0, 100 mM NaCl, 0.1% Triton X-100, 300 mM sucrose, 3 mM MgCl_2_, 5 mM Na_3_VO_4_, 5mM β-glycerophosphate disodium; 1× *Phosphatase Inhibitor Cocktail 3 (Sigma)*; 3× protease inhibitor cocktail (Roche)). After incubation on ice for 10 min, cells were centrifuged (5 min; 3000 × *g*; 4°C) and the supernatant was saved (Soluble I). The treatment with the extraction buffer was repeated with the precipitated material. Then, the suspension was centrifuged again and the supernatant was saved as soluble fraction (Soluble II). The precipitated material was treated with DNaseI (Thermo Fisher) (0.1U/µl) for 25 min at room temperature. The material was centrifuged (5 min; 5000 × *g*; 4°C) and the supernatant was collected (Chromatin).

### EdU incorporation and quantification

Cells were incubated with 10 μM of EdU (Click-iT EdU Image Kit; Thermo Scientific) for 1 h. Cells were fixed with 3.7% paraformaldehyde for 15 min, adhered into poly-L-lysine coated slides and then permeabilized with 0.5% TritonX100 for 20 min. Cells were washed with PBS containing 3% BSA and then subjected to Click-iT reaction for 30 min at room temperature. DNA was stained with Hoechst 33342. Quantification of EdU fluorescence intensity was performed with ImageJ software.

## RESULTS

### HUS1 is essential for *L. major* survival

**Figure 1.**
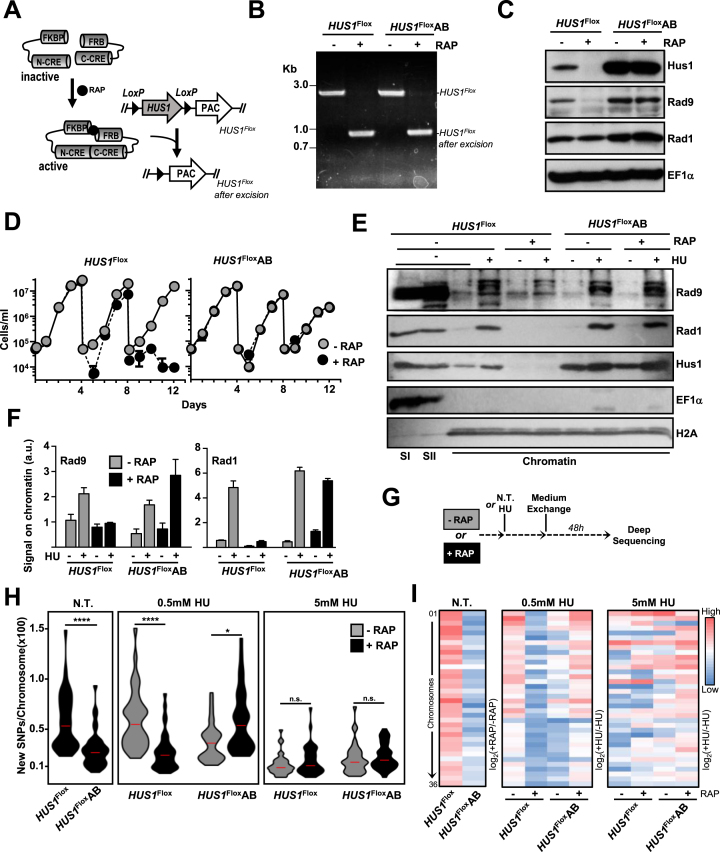
HUS1 in required for genome stability in *Leishmania*. (**A**) DiCre-based system for inducible KO: Cre recombinase is expressed as two inactive truncated forms fused with FKBP and FRB; RAP addition promotes dimerization and reconstitution of the recombinase, which catalyzes the excision of *LoxP* flaked (Flox) sequences. (**B**) PCR analysis of genomic DNA from *HUS1*^Flox^ and *HUS1*^Flox^AB cells after 72 h of RAP induction (first round of induction; see induction strategy in Supplementary Figure S2b and c); primers used were *OL-5UTR (HUS1)* and *OL-15* (Supplementary Figure S1b). (**C**) Western blotting analysis of whole cell extracts from *HUS1*^Flox^ and *HUS1*^Flox^AB cells after 6 days of RAP induction (48 h of second round of induction; see induction strategy in Supplementary Figure S2b and c); extracts were sequentially probed with the indicated antibodies; EF1α was used as loading control. (**D**) Representative growth curves of *HUS1*^Flox^ and *HUS1*^Flox^AB cell lines in the presence or absence of RAP; cells were seeded at ∼5 × 10^4^ cells/ml in day 0 and re-seeded every 4 days to complete three rounds of induction (day 0–4: first round; day 4–8: second round; day 8-9: third round); cell density was assessed every 24 h and error bars depict standard deviation (SD). (**E**) Western blot analysis of chromatin enriched fractions obtained from cells after 48 h of RAP induction; cells were left untreated or incubated with 5 mM HU for 5 h prior to fractionation; fractions were sequentially probed with the indicated antibodies; SI and SII indicate soluble fraction I and II, respectively, and serve as demonstration of fractionation efficiency; chromatin indicates fractions containing proteins released upon DNAseI treatment (see *Experimental Procedures* section for details on the protocol); EF1α was used as a marker for the soluble protein-containing fractions; histone H2A was used as a marker for the chromatin-containing fractions and also as a loading control. (**F**) Quantitative analysis of Rad9 (left) and Rad1 (right) levels on chromatin, from two independent experiments; signal is expressed as arbitrary units (a.u.) relative to H2A signal; error bars depict standard error of the mean (SEM). (**G**) Schematic 1-4representation of treatments prior collection of DNA for deep sequencing: after 48 h of RAP induction, cells were left untreated (N.T.) or treated with 0.5 mM HU (∼20 h) or with 5 mM HU (∼10 h); HU was removed and cells were allowed to proliferate further in HU-free medium for 48 h; then, genomic DNA was prepared and subjected to deep sequencing (Illumina). (**H**) Quantification of numbers of new SNPs per chromosome after RAP induction and/or HU treatments, as indicated; data are represented as violin plots, where shape indicates the distribution of pooled data and horizontal red lines indicate median; differences were tested with Kruskal–Wallis test and are as indicated: (*), 0.0145; (****), <0.0001; n.s., not significant (**I**) Heatmap of SNP enrichment per chromosome (horizontal lines); the data are represented as log2 of the ration of the number of SNPs between the indicated conditions; color scale indicates low (blue) to high (red) SNP enrichment; scale was set individually for N.T., 0.5 mM HU and 5 mM HU groups.

To comprehensively investigate the HUS1 function in *L. major*, we generated a *HUS1*^Flox^ cell line in which expression of DiCre allows for conditional knockout (KO) of *HUS1* flanked by *LoxP* sites upon rapamycin (RAP) induction (19) (Figure 1A–C). An add-back cell line (*HUS1*^Flox^AB), overexpressing HUS1 from an episome, was also generated to control for off-target phenotypes following KO. The genetic makeup of *HUS1*^Flox^ and *HUS1*^Flox^AB cells (Supplementary Figure S1a–c), as well as a time-course of *HUS1* excision (Supplementary Figure S2a), was evaluated by PCR. After 4 days of RAP induction, *HUS1*^Flox^ cells presented substantial loss of HUS1 protein and, following a second round of induction, no protein was detectable (Figure 1C; also see induction strategy in Supplementary Figure S2b and c). After 8 days of induction, cells showed impaired proliferation (Figure 1D), suggesting that, rather than an immediate effect on survival, absence of HUS1 has a cumulative effect, probably due to progressive accumulation of DNA damage or mutations in the genome. HUS1 abrogation also led to a dramatic decrease in RAD9 levels (Figure 1C and Supplementary Figure S4c), defective chromatin association of RAD9 and RAD1 upon replication stress (Figure 1E and F), and heightened sensitivity to replication stress-causing agents, such as hydroxyurea (HU), camptothecin (CPT) and methyl methanesulfonate (Supplementary Figure S3a–d). The use of a cell line expressing RAD9 from a transfected episome (*HUS1*^Flox^ *RAD9*) prevented the drastic decrease in RAD9 levels upon RAP induction but did not rescue the dramatically impaired cell growth caused by HUS1 abrogation (Supplementary Figure S4a–d). These data demonstrate that *HUS1* is pivotal for the replication stress response and also plays essential roles in *L. major* promastigotes that cannot be supplied by RAD9 and/or RAD1 alone.

### HUS1 is required for genome stability under non-stressed conditions, but promotes genome variability under chronic replication stress

To understand the contribution of HUS1 to genome maintenance in *L. major*, we analyzed the genome instability in HUS1-depleted cells by determining genome-wide accumulation of SNPs, which may result from distinct defects in DNA repair and replication (Figure 1G). In virtually every chromosome RAP induction led to increased SNP accumulation in *HUS1*^Flox^ cells compared to *HUS1*^Flox^AB cells under non-stressed conditions, demonstrating that loss of HUS1 had a genome-wide mutagenic effect (Figure 1H and I). We further analyzed SNP accumulation upon replication stress using two treatments: 0.5 mM HU for a longer period of time (chronic replication stress); or 5 mM HU for a shorter period of time (acute replication stress). In uninduced cells, higher numbers of new SNPs were observed after chronic replication stress compared with acute treatment (Figure 1H). Surprisingly, loss of HUS1 resulted in decreased SNP accumulation after chronic replication stress compared to non-induced or add-back cells (Figure 1H and I). In contrast, HUS1 ablation resulted only in a marginal increase of new SNPs after acute replication stress (Figure 1H and I). It is noteworthy that A, T, C and G nucleotide residues seemed to be mutated at comparable proportions in all conditions analyzed (Supplementary Figure S5a). Also, *HUS1* loss did not lead to any significant trend toward either transitions or transversions among SNP events (Supplementary Figure S5b). Analysis of genome-wide accumulation of structural variations also yielded similar results (Supplementary Figure S6), further evidencing HUS1 involvement in genome maintenance. These results reveal a number of things. First, chronic replication stress promotes higher genomic instability than acute stress. Second, HUS1 appears dispensable for genome stability under acute stress. Third, and counterintuitively, whereas loss of HUS1 increases genome instability during normal growth, loss of the factor enhances stability during chronic replication stress. Thus, HUS1 may provide variant functions after different replication stress treatments, and so we set out to investigate the cellular consequences of HUS1 absence in these conditions.

### The distinct roles of HUS1 are associated with different patterns of ssDNA accumulation

**Figure 2.**
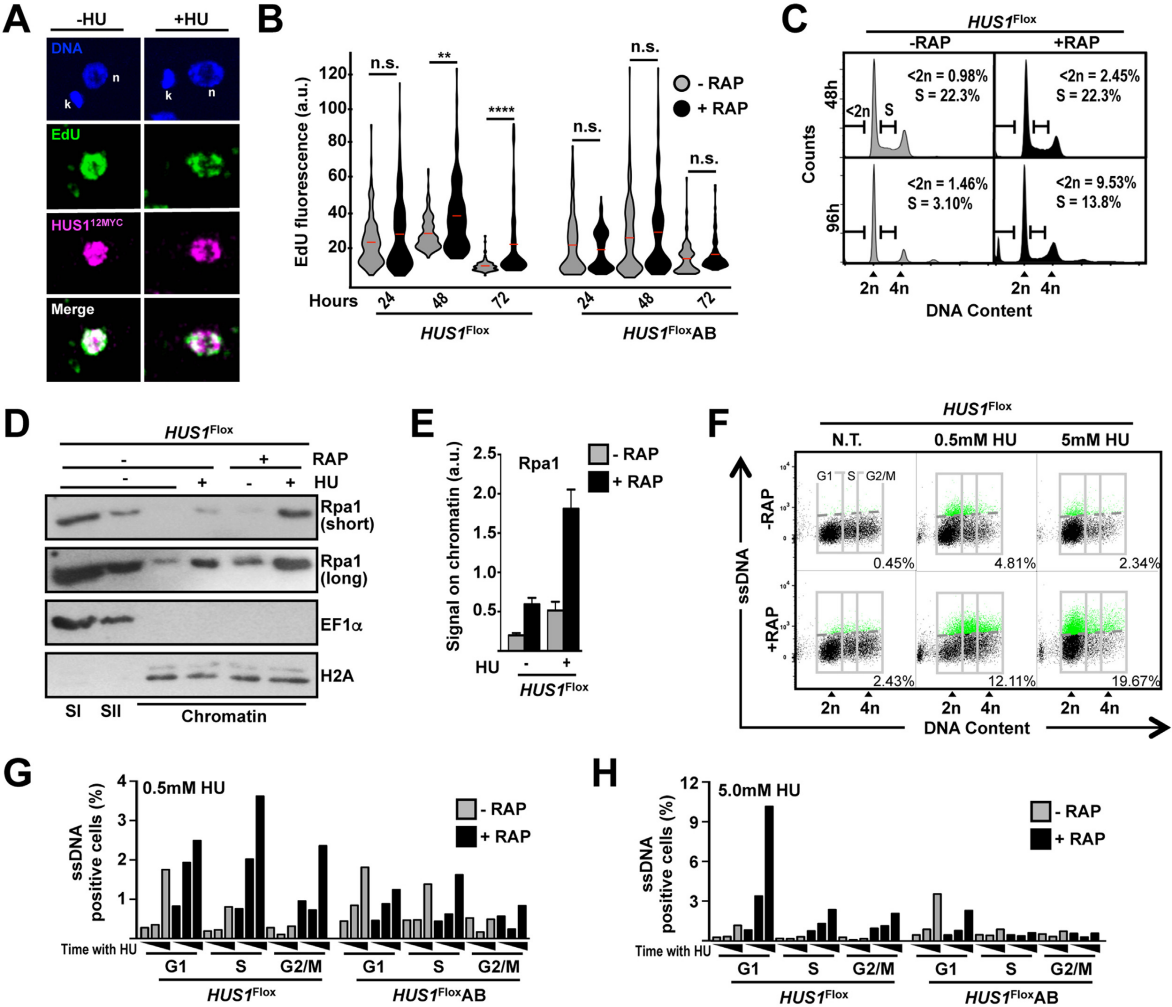
Distinct requirements for HUS1 in preventing single-stranded DNA accumulation upon chronic and acute replication stress. (**A**) Co-localization of HUS1 and replication foci; cells expressing HUS1-12xMYC from the endogenous locus (17) were left untreated (−HU) or treated with 5 mM HU (+HU) for 5 h; HU was removed and cells were incubated with 10 mM EdU for 1 h, fixed and processed for sequential EdU and HUS1-12xMYC detection; images correspond to a single confocal Z-slice (∼0.13 μm thickness) after deconvolution using BlindDblur (LAS AF Leica Software); n and k indicate nuclear and kinetoplast DNA, respectively. (**B**) Quantitative analysis of EdU following induction of *HUS1* KO; at the indicated time after RAP induction, cells were labeled with 10 μM EdU for 1 h and processed as in (A); EdU fluorescence is expressed as arbitrary units (a.u.); three independent experiments were performed and at least 300 cells were analyzed in each time point; data are represented as violin plots, where shape indicates the distribution of pooled data and horizontal red lines indicate mean; differences were tested with Kruskal–Wallis test and are as indicate: (**), *P* = 0.0033; (****), *P* < 0.0001; n.s., not significant. (**C**) Representative histograms from FACS analysis to determine the distribution of cell population according to DNA content at the indicated time points after RAP induction; 50 000 cells were analyzed per sample; 2n and 4n indicate non-duplicated (G1) and duplicated (G2/M) DNA, respectively; <2n and S represent gates used to determine the percentage of cells with DNA content less than the diploid content and S-phase cells, respectively; quantification of the proportion of these two population throughout the course of RAP induction is shown in Supplementary Figure S8. (**D**) Western blot analysis of chromatin-enriched fractions as in Figure 1E; short and long indicate relative exposure time of membrane to film. (**E**) Quantitative analysis of RPA1 levels on chromatin; analysis was performed as in Figure 1F; three independent experiments were used for the analysis; error bars depict SEM. (**F**) Representative dot plot from FACS analysis to determine distribution of ssDNA-positive cells through cell-cycle phases: 36 h after RAP induction, cells were incubated with IdU for 14 h; then, cells were left untreated (N.T.) or incubated with 0.5 mM HU (20 h) or 5 mM HU (8 h); detection of IdU under non-denaturing conditions was used for ssDNA measurement; 2n and 4n indicate non-duplicated (G1) and duplicated (G2/M) DNA, respectively; gray boxes indicate gates used to determine proportion of cells with DNA content corresponding to G1, S and G2/M, as indicated; dotted gray lines indicate threshold to discriminate negative (black dots) from ssDNA-positive (green dots) events (see gate strategy in Supplementary Figure S11); inset numbers indicate total percentage of ssDNA-positive events relative to the whole population; 50 000 cells were analyzed per sample; see extended data in Supplementary Figure S12. ( **G** and **H**) Quantitative analysis of ssDNA-positive cell distribution among cell-cycle phases upon the indicated HU treatment; after addition of 0.5 mM HU, cells were collected at 0, 8 and 20 h; after addition of 5 mM HU, cells were collected at 0, 4 and 8 h; data represent the mean from two independent experiments;

Confocal microscopy showed substantial co-localization of endogenously tagged HUS1 and newly synthesized DNA (as measured by EdU incorporation) in unstressed and, to a lesser extend, in HU-treated cells (Figure 2A and Supplementary Figure S7), consistent with the protein being involved in DNA synthesis. As was observed in analysis of *HUS1+/-* cells (17), EdU incorporation was significantly increased upon HUS1 ablation (Figure 2B), which demonstrates that, at least in unperturbed cells, HUS1 affects DNA synthesis, perhaps as an intra-S checkpoint factor, as previously suggested (17,18). In line with this, FACS analysis showed that HUS1 loss resulted in a higher proportion of cells in S-phase (Figure 2C and Supplementary Figure S8a). Also, we found that levels of the ssDNA binding protein RPA1 increased in chromatin fractions of *HUS1* ablated cells, an effect that was increased further after HU-treatment (Figure 2D and E). To explore this effect further, we directly probed the generation of ssDNA by IdU detection under non-denaturing conditions (Supplementary Figure S9a–c). Microscopy (Supplementary Figure S10a and b) and FACS (Figure 2F) analysis showed that ssDNA accumulation in *HUS1* KO cells was dramatically increased upon both chronic and acute HU replication stress. Next, we used the FACS analysis to determine the distribution of ssDNA-positive cells throughout the cell-cycle phases (Supplementary Figure S11a). HUS1 absence led to an overall increase in the number of ssDNA-positive cells, in both non-stressed and HU-stressed conditions, in all phases of the cell cycle (Figure 2F and Supplementary Figure S12). However, treatment of *HUS1* KO cells with chronic replication stress resulted in a more prominent increase of ssDNA-positive cells in S and G2/M, whereas upon acute stress the majority of cells with increased ssDNA were in G1 (Figure 2G and H). Taken together, these data suggest two things. First, loss of HUS1 in unstressed cells leads to increased or unscheduled DNA synthesis, leading to elevated levels of ssDNA, perhaps explaining the genome-wide SNP accumulation. Second, depending on the level of exogenous replication stress, HUS1 contributes to the control of replication either at early S-phase or progression through S phase. Therefore, the distinct effects of *HUS1* KO on SNP accumulation upon each type of treatment can be explained, at least partially, by the distinct profile of ssDNA accumulation resulting from each treatment.

### HUS1 modulates mitosis onset and DNA damage signaling during G2/M checkpoint

**Figure 3.**
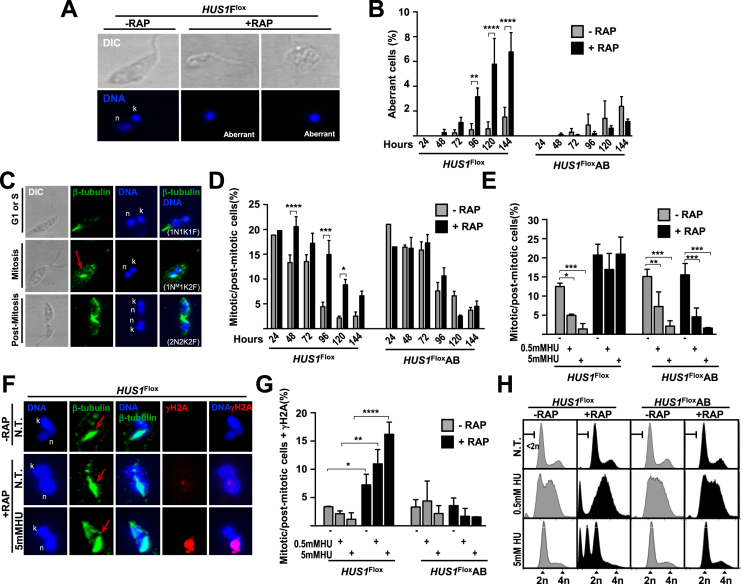
HUS1 is required for the G2/M checkpoint and for establishment of distinct cell-cycle arrest and progression after chronic and acute replication stress. (**A**) Representative image showing *HUS1*^Flox^ cells 144 h of RAP induction with aberrant DNA configuration; these cells do not present the normal DNA configuration comprising one nuclear and one kinetoplast compartment. (**B**) Quantification of the proportion of cells with aberrant DNA configuration relative to total number of cells, at the indicated time points after RAP induction; three independent experiments were performed and at least 130 cells were analyzed in each time point; differences were tested with one-way ANOVA test and are as indicate: (**), *P* = 0.0065; (****), *P* < 0.0001; error bars indicate SEM. (**C**) Representative image from immunofluorescence analysis of β-tubulin distribution pattern in *HUS1*^Flox^ cells in exponential growth; red arrow indicates a characteristic mitotic spindle found in cells undergoing mitosis; n and k indicate nuclear and kinetoplast DNA, respectively; number of nucleus (N), kinetoplast (K), flagellum (F) and the presence of mitotic spindle (^M^) can serve as indication of nuclear division; 1N1K1F, cells in G1 or S phase; 1N^M^1K2F, cells undergoing mitosis; 2N2K2F, post-mitotic cells. (**D**) Quantification of the proportion of cells with mitotic (1N^M^1K2F) or post-mitotic (2N2K2F) configurations relative to total number of cells, at the indicated time points after RAP induction; two independent experiments were performed and at least 180 cells were analyzed in each time point; differences were tested with one-way ANOVA test and are as indicate: (*), 0.0217; (***), 0.0007; (****), 0.0006; error bars indicate SEM. (**E**) Quantification of the proportion of cells with mitotic (1N^M^1K2F) or post-mitotic (2N2K2F) configurations relative to total number of cells; three independent experiments were performed and at least 300 cells were analyzed for each condition; differences were tested with one-way ANOVA test and are as indicate: (*), *P* = 0.0532; (**), *P* = 0.0462; (***), *P*< 0.01; error bars indicate SEM. (**F**) Representative image from immunofluorescence analysis of β-tubulin and γH2A: 48 h after RAP induction, cells were left untreated (N.T.) or treated with HU; red arrow indicates mitotic spindle; n and k indicate nuclear and kinetoplast DNA, respectively; In (A), (C) and (F), images correspond to a Z-projection from serial acquisition with Confocal Leica TCS SP5 microscopy. (**G**) Quantification of the proportion of cells with γH2A signal and mitotic (1N^M^1K2F) or post-mitotic (2N2K2F) configurations relative to total number of cells; three independent experiments were performed and at least 300 cells were analyzed for each condition; differences were tested with one-way ANOVA test and are as indicate: (*), *P* = 0.0253; (**), *P* = 0.0041; (****), *P*< 0.0001; error bars indicate SEM. (**H**) Representative histograms from FACS analysis to determine the distribution of the cell population according to DNA content: 48 h after RAP induction, cells were left untreated (N.T.) or treated with 0.5 mM HU (20 h) or 5 mM HU (8 h), fixed and subjected to analysis; 50 000 cells were analyzed per sample; 2n and 4n indicate non-duplicated (G1) and duplicated (G2/M) DNA, respectively; <2n represents a population of cells with DNA content less than the haploid content.

The involvement of the 9–1–1 complex in the G2/M checkpoint seems to be conserved among eukaryotes (27–29). A defective G2/M checkpoint can lead to premature nuclear division, resulting in cells with <2n DNA content and/or aberrant DNA configurations. Consistent with this, we observed accumulation of cells with <2n DNA content (Figure 2C and Supplementary Figure S8b) and aberrant DNA configurations (Figure 3A and B) upon *HUS1* KO. To further explore if this phenotypes was co-related with defective G2/M checkpoint, we quantified the proportion of cells with mitotic or post-mitotic β-tubulin and DNA configurations (30). HUS1 ablation caused a significant increase in cells with these configurations, demonstrating that HUS1 controls the onset of mitosis in cells even in the absence of exogenous stresses (Figure 3C and D). We also performed this analysis in cells subjected to chronic and acute HU-derived replication stress. While both stress conditions drastically reduced the proportion of mitotic or post-mitotic cells in non-induced cultures, no significant reduction was observed in HUS1 ablated cells (Figure 3E). These findings support a role for HUS1 in the G2/M checkpoint in both not-treated and replication stressed conditions. We next quantified the proportion of cells with mitotic or post-mitotic configurations simultaneously bearing phosphorylated histone H2A (γH2A), a DNA damage marker in trypanosomatids (17,31) (Figure 3F). We found that upon HUS1 KO there was a significant increase in the proportion of this type of cells in both not-treated and replication-stressed conditions (Figure 3G). Thus, these data show that in the absence of HUS1 there is increased mitosis onset in the presence of genome injuries, suggesting that HUS1’s role in the G2/M checkpoint is to prevent entry into mitosis in the presence of DNA damage.

### The distinct roles of HUS1 are associated with different patterns of cell-cycle progression after replication stress

In order to understand the effect of HUS1 loss on cell-cycle progression and in damage repair, we next used FACS to analyze cell-cycle distribution (Figure 3H). HUS1 ablation resulted in massive accumulation of <2n cells upon both types of replication stress, further evidencing its involvement in the G2/M checkpoint. Treatment of non-induced cells with chronic stress resulted in a diffuse distribution across the cell-cycle phases, suggesting that, if replication is engaged, DNA synthesis proceeds at a lower rate. On the other hand, S-phase progression seems to be completely prevented upon acute stress, since cells accumulated at the G1/S border (Figure 3H). *HUS1* KO cells were considerably slower than uninduced cells in cell-cycle progression after release from chronic replication stress (Supplementary Figure S13a), whereas a faster progression of KO cells was observed after release from acute treatment (Supplementary Figure S13b). Altogether, these data demonstrate a pivotal role of HUS1 in the establishment of specific patterns of cell cycle arrest and navigation upon chronic and acute replication stress, correlating with the distinct levels of genome variability under these different treatments.

### The distinct roles of HUS1 are associated with different patterns of DNA damage accumulation and resolution after replication stress

**Figure 4.**
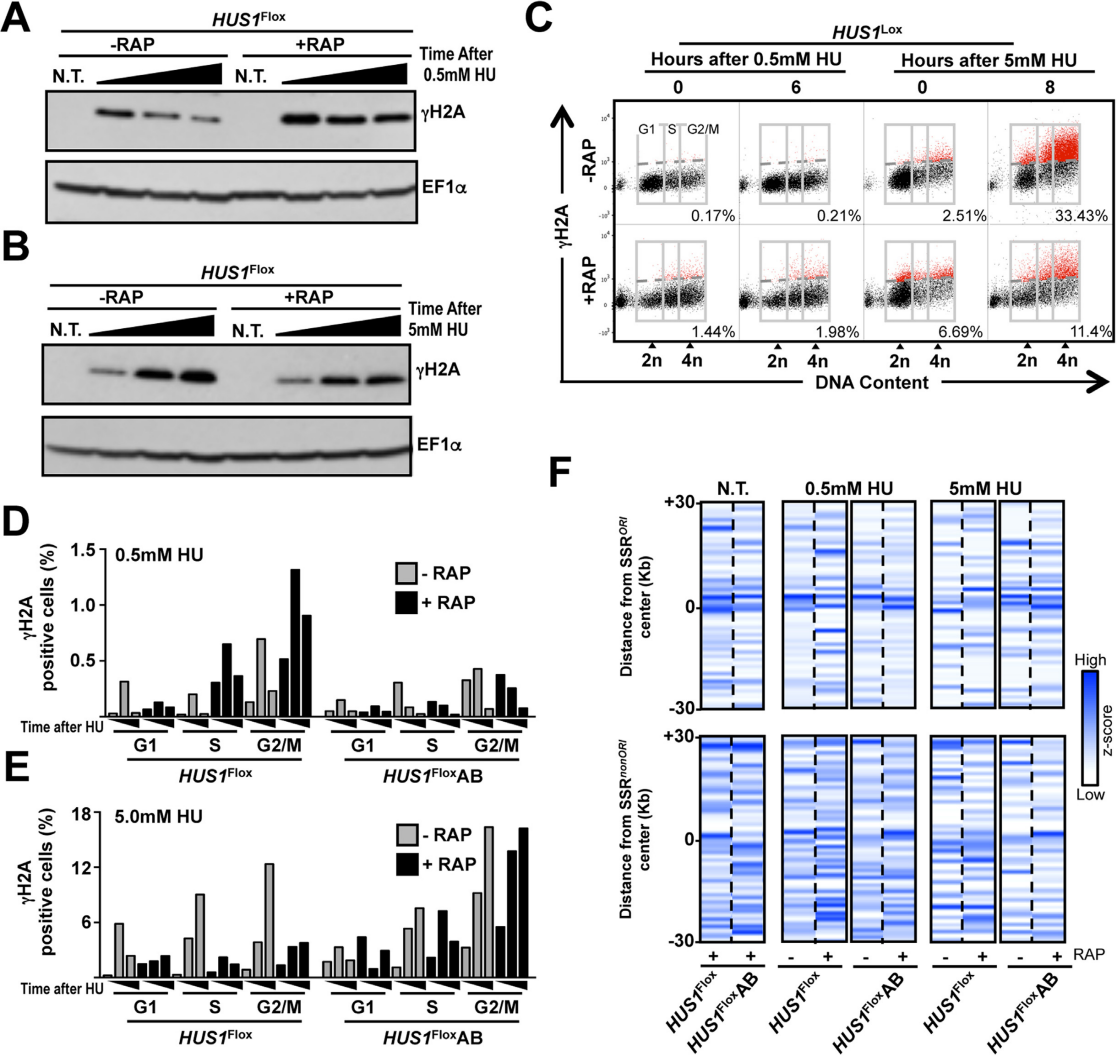
Dual roles of HUS1 dictate distinct DNA damage signaling and genomic instability outcomes after chronic and acute replication stress. ( **A** and **B**) Western blot analysis of whole cell extracts; 48 h after RAP induction, *HUS1*^Flox^ cells were left untreated (N.T.) or treated with 0.5 mM HU (20 h in A) or with 5 mM HU (8 h in B); HU was removed and cells were allowed to proliferate in fresh medium; in (A), cells were collected at 0, 2 and 5 h after HU removal; in (B); cells were collected at 0, 4 and 8 h after HU removal; extracts were probed for γH2A levels and EF1α was used as loading control; an independent experiment is shown in Supplementary Figure S14a and b. (**C**) Representative dot plot from FACS analysis to determine distribution of γH2A-positive cells through cell-cycle phases; 48 h after RAP induction, cells were incubated with 0.5 mM HU (20 h) or 5 mM HU (8 h); HU was removed and cells were allowed to proliferate in fresh medium; cells were collected and subjected to FACS analysis at the indicated time points; 2n and 4n indicate non-duplicated (G1) and duplicated (G2/M) DNA, respectively; gray boxes indicate gates used to determine proportion of cells with DNA content corresponding to G1, S and G2/M, as indicated; dotted gray lines indicate threshold to discriminate negative (black dots) from positive (green dots) γH2A-positive events (see gate strategy in Supplementary Figure S11); inset numbers indicate total percentage of γH2A-positive events relative to whole population; 50 000 cells were analyzed per sample; see extended data in Supplementary Figure S14c. (**D** and **E**) Quantitative analysis of γH2A-positive cells distribution among cell-cycle phases upon the indicated HU treatment: after removal of 0.5 mM HU, cells were collected at 0, 3 and 6 h; after removal of 5 mM HU, cells were collected at 0, 4 and 8 h; data represent the mean from two independent experiments. (**F**) Genome-wide analysis of SNP distribution pattern around SSRs in non-stressed and in replication stressed cells; the *Leishmania major* genome was binned in 1 Kb windows and SNP occurrence in each bin was determined in terms of *z*-scores; chromosomes were aligned around either SSR*^ORI^* (*n* = 36) or SSR*^non-ORI^* (*n* = 95) regions; *z*-score mean was calculated for every bin in 30 Kb around SSR centers and normalized before plotting; color scale indicates low (white) to high (blue) *z*-score; scale was set individually for N.T., 0.5 mM HU and 5 mM HU groups.

To understand how HUS1 participates in DNA damage signaling or repair during the above responses, we examined γH2A levels after exposure to chronic or acute replication stress. In the absence of RAP induction, both HU treatments resulted in increased γH2A signal, but this diminished relatively quickly after chronic stress and persisted after acute stress (Figure 4A and B; Supplementary Figure S14a and b). Again, the effect of HUS1 loss differed in the two conditions. Consistent with delayed cell cycle progression, *HUS1* KO cells presented persistent γH2A levels after removal of chronic replication stress (Figure 4A and Supplementary Figure S14a). On the other hand, *HUS1* KO cells showed a decrease in the persistent γH2A levels after removal of acute replication stress (Figure 4B and Supplementary Figure S14b), correlating with the faster cell-cycle progression. FACS analysis confirmed the western blotting and showed that HUS1 loss led to an increased proportion of γH2A-positive cells after chronic replication stress compared with uninduced cells, while a decreased proportion of positive cells was observed after acute stress (Figure 4C and Supplementary Figure S14c). Quantification of the cell-cycle distribution of the γH2A-positive cells showed that in uninduced cells, in both treatments, most γH2A signal was in S or G2/M cells. After HUS1 loss and chronic stress the persistent γH2A signal was also most clear in S and G2/M cells, whereas there was a marked reduction in γH2A accumulation in these cell-cycle stages after acute stress (Figure 4D and E). These findings indicate that the different replication stresses result in distinct patterns of DNA damage signaling throughout the cell cycle and that HUS1 is pivotal to the establishment of such patterns.

### HUS1 is required for the establishment of genome instability patterns upon chronic replication stress

The data presented above suggest HUS1 contributes to nuclear DNA replication fidelity. To test this further, we inquired into the effect of each type of replication stress on genome stability, with and without HUS1 loss, by examining the pattern of SNP appearance in relation to DNA replication origins. In *L. major*, replication mapping by MFA-seq indicates that DNA replication initiation occurs predominantly at a single origin in each chromosome, with each origin co-localizing with so-called switch strand regions (SSRs) between directional gene clusters (32,33). Thus, we mapped the pattern of SNP distribution in the vicinity of all origin-containing SSRs (SSR*^ORI^*) and all SSRs that do not display origin activity (SSR*^nonORI^*). We found that SNP accumulation was concentrated around the SSR*^ORI^* center (Figure 4F, upper panels), while no such pattern was observed around SSR*^nonORI^*(Figure 4F, lower panels). The SNP accumulation pattern around SSR*^ORI^* was visible in all the RAP-induced *HUS1*^Flox^ and add-back cells, with or without HU treatment, with one exception. In HUS1-depleted cells after chronic stress, SNPs did not collect at the centre but were distributed more evenly around of the SSR*^ORI^*. This observation reflects the consistent differences observed in DNA damage signaling, cell-cycle progression and ssDNA formation due to chronic or acute replication stress dictated by HUS1. Thus, the pattern of mutagenesis around SSR*^ORI^* reflects the predominance of bi-directional replication from single origins. One explanation may be increased collision rate or severity between the replication and transcription machineries due to constitutive replication from SSR*^ORI^*, an effect not seen at SSR*^nonORI,^* where replication either does not initiate or initiates infrequently. Also, these data firmly link HUS1 function in the response to chronic replication stress with the unusual DNA replication dynamics of *Leishmania*.

## DISCUSSION

Evaluation of the roles of essential gene products in *Leishmania* has been burdensome until the recent development of DiCre-mediated conditional gene deletion (19). Here, we have used this approach to explore what roles are played by HUS1, the first *Leishmania* genome maintenance factor examined in this way. Our findings advance our understanding of HUS1 function and mode of action in controlling genome stability. Also, the observation that HUS1 promotes genome variability indicates the existence of unusual genome maintenance processes in this protozoan parasite.

The detrimental effect on parasite growth observed upon HUS1 abrogation suggests that the protein is essential for *L. major*. Similar effects were also observed upon HUS1 depletion in mammal cells (34–36), while other eukaryotes are able to survive without HUS1 (37). In the absence of HUS1, the 9–1–1 complex seems to be unable to properly operate, given the dramatic reduction of RAD9 levels, the defective chromatin association of both RAD9 and RAD1, and increased sensitivity to replication stress. Our data indicate that HUS1 function is centred around at least two axes. One of these axes is the conserved role of the 9–1–1 complex in the G2/M checkpoint seen potentially in all eukaryotes (27,38). Until now, we did not know if and how HUS1 contributes to this checkpoint in *L. major*. Here, we show that in both normal and replication-stressed conditions, HUS1 acts by modulating the onset of mitosis through preventing inappropriate nuclear division in the presence of genome injuries. The other axis of HUS1 action is during the DNA replication process. We have previously observed that HUS1 is involved in *L. major* DNA replication (17), but its mode of action was unclear. Here we demonstrate that HUS1 co-localizes with replication foci, further evidencing its involvement with the replication process in this parasite, though whether this role is solely in response to DNA damage or may be more extensive needs further analysis. The less prominent co-localization of HUS1 with EdU in HU-treated cells suggests that HUS1 function in this condition is predominantly detached from the replication fork. The EdU incorporation analysis confirmed, in a time-course-manner, the requirement of HUS1 to negatively modulate DNA replication in non-stressed conditions. Furthermore, the accumulation of RPA-1 on chromatin and ssDNA upon induction of *HUS1* KO suggest that the role of HUS1 in DNA replication is related with the resolution of endogenous replication stress. Whether this replication stress is the cause or consequence of the deregulated DNA synthesis upon *HUS1* KO is yet to be determined. It is not clear which of the above HUS1 functions are required for parasite survival. Possibly, the combination of many effects after KO induction underlies *HUS1*’s essentiality.

By using chronic and acute replication stress treatments we were able to demonstrate that this protozoan has evolved the ability to coordinate distinct responses to different levels of genotoxic stress. We also show that HUS1 participates in gauging different levels of replication stress by dictating distinct molecular and cellular outcomes upon chronic and acute replication stress. HUS1 absence is associated with increased genome instability in unstressed cells and also upon acute replication stress, albeit to a lesser extent. These findings agree with the role of the 9–1–1 complex as a contributor to genome stability as described in other eukaryotes (14–16). Whether *Leishmania* HUS1’s contribution to genome stability is related to replication fork stabilization, DNA repair or telomere maintenance is still unclear. On the other hand, HUS1 abrogation led to lowered levels of genomic variability in cells exposed to chronic replication stress, which contrasts with its conserved role in safeguarding genome maintenance. We did not observe any significant change in chromosome copy number upon *HUS1* KO or HU treatments (data not shown). Possibly, the narrow time window in which *HUS1* KO cells were viable and during which the experiments were performed, was not enough to allow cells with altered ploidy to take over the population to a detectable level. Nonetheless, it remains to be examined in the future if and how any of the 9–1–1 complex subunits contributes to the ploidy maintenance in *Leishmania*

In both acute and chronic replication stress, the G2/M checkpoint is similarly affected by HUS1 abrogation, suggesting that aberrant nuclear division does not dictate the different outcomes in genome stability between the two treatments. On the other hand, we did observe substantial differences in the pattern of ssDNA accumulation between the two treatments in *HUS1* KO cells. Upon chronic or acute replication stress, control cells that accumulate ssDNA are retained at the G1 phase of the cell cycle. Such retention, possibly at the early steps of DNA replication, is lost in *HUS1* KO cells exposed to chronic replication stress, which correlates with the decrease in genome variability. Perhaps, under chronic replication stress HUS1-dependent genome variability is confined to the early-S phase. Consistent with this, the accumulation of SNPs around early-S origins is lost in *HUS1* KO cells under chronic replication stress. Together, these findings support a cell cycle-dependent link between HUS1, or the entire 9–1–1 complex, and genomic variability when exposed to chronic replication stress. Although speculative, the dispersed SNP pattern around early-S origins in *HUS1* KO cells under chronic replication stress also suggests a functional connection between HUS1 and DNA replication initiation. As early-S origins are also sites of transcription initiation, it remains to be tested if HUS1’s function in promoting genomic variability is related to interaction between DNA replication and transcription. Collisions between transcription and replication machineries are known sources of genome instability (39–41). Thus, it will be necessary to determine if the 9–1–1 complex contributes to recognizing, signaling or resolving collisions between replication and transcription in *Leishmania*.

We show that DNA damage signaling and resolution, as measured by γH2A levels, varies not only between chronic and acute replication stress, but is also affected by HUS1 expression. This suggests that the distinct effects of HUS1 abrogation in genomic stability depends on the resolution of DNA damage after replication stress. Decreased DNA damage signaling may explain the faster cell-cycle progression in *HUS1* KO cells after acute replication stress, which would cause mutations to accumulate. On the other hand, upon chronic replication stress, HUS1 expression is associated with faster cell-cycle progression and decreased DNA damage signaling. A speculation is that, under these conditions, HUS1 participates in the re-engagement of replication, thus allowing for the cell cycle to resume correctly. However, under chronic replication stress, resuming replication would come at the cost of increased mutagenesis.

The 9–1–1 complex has been implicated in DNA damage tolerance (DDT) through both error-free and error-prone pathways (42–45). Error-prone DDT pathways modulated by the 9–1–1 complex have been characterized in the response to alkylating-, crosslinking- or UV-derived DNA damage and require translesion synthesis (TLS) catalyzed by low fidelity DNA polymerases (44,45). These enzymes bypass DNA lesions and allow the restart of halted DNA replication forks, frequently being a source of mutagenesis (46). Here we show that HUS1 expression correlates with increased genomic variability in cells exposed to HU-induced chronic replication stress. Although this type of genotoxic stress does not primarily generate structural damage at the level of the nucleotide residues, there is evidence that some TLS DNA polymerases are required for DNA synthesis upon HU treatment in other eukaryotes (47). However, the HUS1-mediated variability in *Leishmania* did not present any obvious TLS-mediated mutation biases. Nonetheless, it remains to be tested if the genomic variability mediated by HUS1 in *Leishmania* during chronic replication stress requires the recruitment and modulation of any conserved or divergent TLS DNA polymerases.

It is still unclear how the 9–1–1 complex regulates the choice of error-prone or error-free pathways in *Leishmania*. Possibly, in both acute and chronic replication stress, the 9–1–1 complex localizes to stalled DNA replication forks and/or their vicinity, serving as a scaffold to recruit signaling and repair factors. Perhaps the factors recruited, or the regulatory modification of the 9–1–1 subunits, as seen in other eukaryotes (48), may differ between the two conditions, either due to differences in the extension of replication stress and cell-cycle position, thus activating distinct replication restart pathways. Also, this functional diversity of HUS1 correlates with the variety of complexes formed by this protein *in vivo* (17). In other eukaryotes, variant subunits of the 9–1–1 complex seem to account for a wide range functions (49,50). We have previously observed that in *Leishmania* HUS1 is present in the context of the 9–1–1 complex and also as a monomer, while RAD9 is found in the context of the 9–1–1 complex and also in a distinct complex, correlating with compartmentalized functions of the two subunits. Our present data suggest that such functional diversity is not restricted to the different subunits. The distinct effects of HUS1 abrogation on genome stability indicate that functional diversity is also present at the level of an individual subunit. Given the existence of distinct forms of HUS1 *in vivo*, it is reasonable to speculate that its distinct forms dictate the functions associated with genome stability and genome variability. Therefore, it is still an open question which form of HUS1 (monomeric or 9–1–1-associated) is required for each of the observed effects. Also, the broader implications of this divergent role of HUS1, especially in the context of infection, need to be further explored. It is possible that, when faced with chronic replication stress within the host cell, HUS1 safeguards genomic variability, increasing survival and ensuring successful infection.

## Supplementary Material

Supplementary Data
