# Supplementary material for: Conditional genome engineering reveals canonical and divergent roles for the Hus1 component of the 9–1–1 complex in the maintenance of the plastic genome of Leishmania
Source: Nucleic Acids Res. 2018 Oct 31;46(22):11835–46. doi: 10.1093/nar/gky1017 (PMC6294564; doi:10.1093/nar/gky1017)
Supplement: Supplementary Data [file gky1017_supplemental_files.pdf]

SUPPLEMENTARY INFORMATION

Conditional genome engineering reveals canonical and divergent roles for the Hus1 component of the 9-1-1 complex in the maintenance of the plastic genome of *Leishmania*.

Jeziel D. Damasceno[1], Ricardo Obonaga[1], Gabriel L. A. SIlva[1] João L. Reis-Cunha[2], Samuel M. Duncan[3], Daniella C. Bartholomeu[2], Jeremy C. Mottram[3,4], Richard McCulloch[3] and Luiz R. O. Tosi[1*].

[1]Department of Cell and Molecular Biology, Ribeirão Preto Medical School, University of São Paulo; Ribeirão Preto, SP, Brazil.

[2]Laboratório de Genômica de Parasitos, Departamento de Parasitologia, Instituto de Ciências Biológicas, Universidade Federal de Minas Gerais, Belo Horizonte, Minas Gerais, Brasil

[3]Wellcome Centre for Molecular Parasitology, Institute of Infection, Immunity and Inflammation, University of Glasgow, United Kingdom

[4]Centre for Immunology and Infection, Department of Biology, University of York, York, United Kingdom

*To whom correspondence should be addressed: Tel: +55 16 33153117; Email: luiztosi@fmrp.usp.br.

Supplementary Figure 1

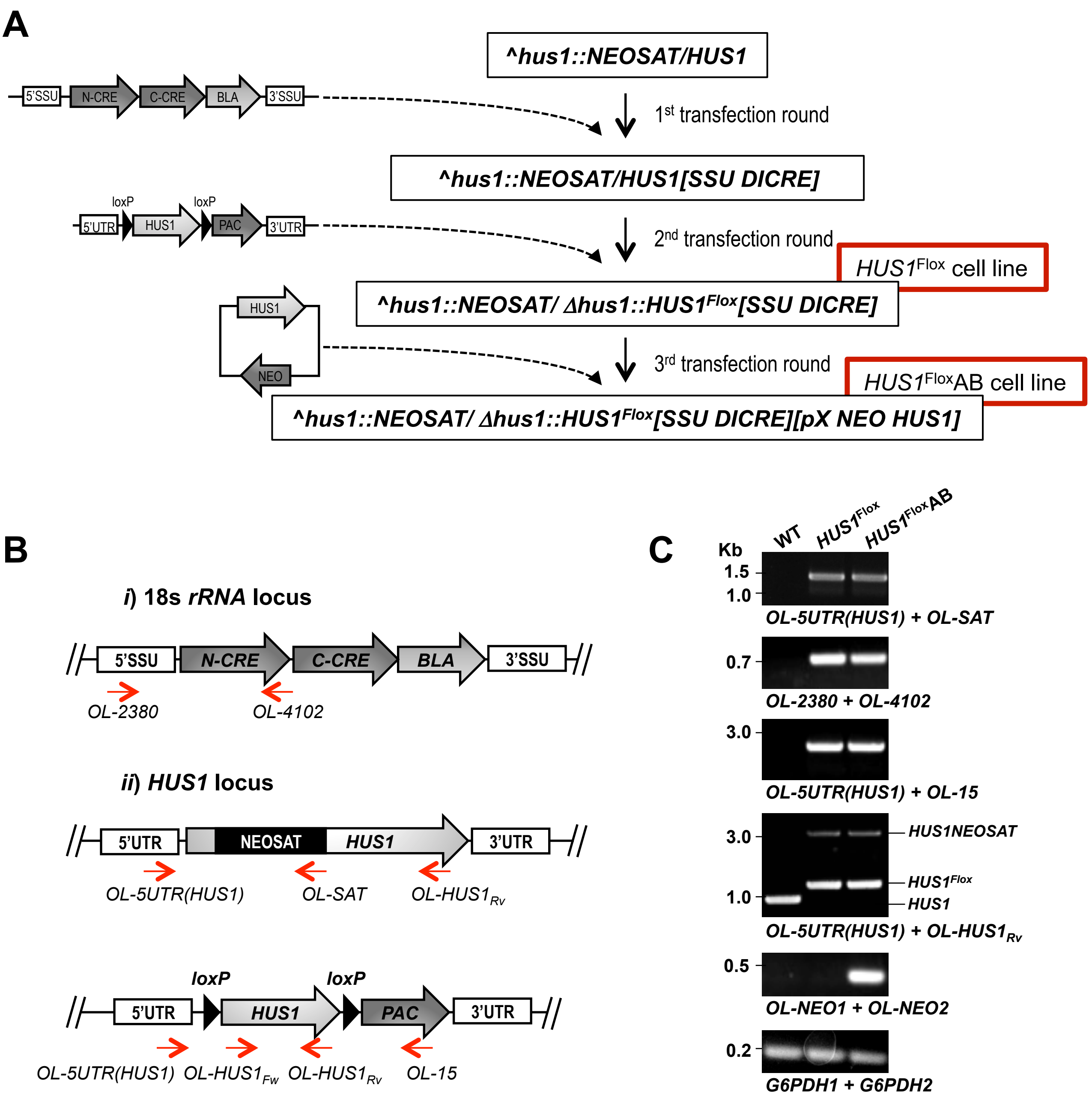

Figure S1. Generation and characterization of ***HUS1*^{Flox} and *HUS1*^{Flox}AB cell lines. A)** Schematic illustration of steps followed for the generation of *HUS1*^{Flox} and *HUS1*^{Flox}AB cell lines; first, HUS1 deficient cell line (*^hus1::NEOSAT/HUS1*) (Damasceno et al., (2013) *Mol Microbiol*, **90**, 1074-1087) was transfected with linear DNA cassette containing the *DICRE* sequence flanked by SSU sequences; second, a clonal cell line resulting from the first step (*^hus1::NEOSAT/ HUS1[SSU DICRE])* was transfected with linear DNA cassette containing the *HUS1* sequence flanked by the loxP sites to generate the *HUS1*^{Flox} cell line (*^hus1::NEOSAT/ Dhus1::HUS1*^{Flox}*[SSU DICRE]*); third, a clonal cell line resulting from the second step was then transfected with a pXG1 NEO plasmid containing the HUS1 coding sequence resulting in the *HUS1*^{Flox}AB cell line. **B)** Representation of the expected genomic configuration of the *DICRE*, *HUS1::NEOSAT* and *HUS1*^{Flox} cassettes in the *HUS1*^{Flox} cell line (not in scale); red arrows indicate approximate annealing position of primers used for PCR analysis shown in (C). **C)** Genomic DNA from the indicated cell lines was PCR amplified using the indicated set of primers; PCR products was resolved in agarose gels and visualized with Ethidium Bromide; OL-NEO1 and OL-NEO2 were used to demonstrate the presence of pXNEOHUS1 plasmid in *HUS1*^{Flox}AB cell line; G6PDH was used as loading control.

Supplementary Figure 2

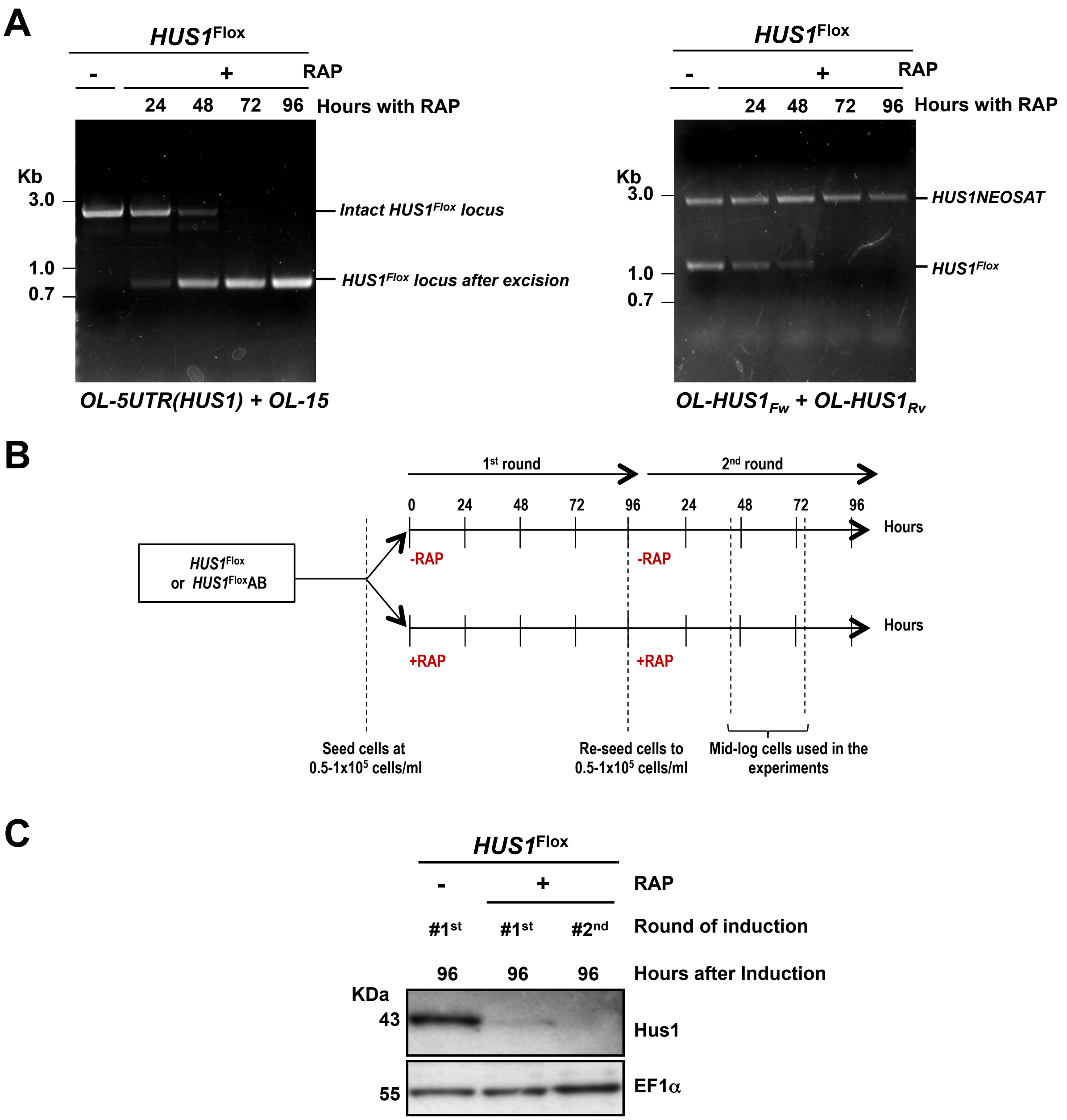

Figure S2. Time course induction of *HUS1* gene excision in the *HUS1*^{Flox} cell line. A) Genomic DNA was extracted at the indicated time points after RAP induction and subjected to PCR analysis using the indicated set of primers (see Supplementary Fig. 1B for annealing position); PCR products were resolved in agarose gels and visualized with Ethidium Bromide. **B)** Illustration of *HUS1* KO induction scheme; cells were seeded in medium with or without RAP; after 4 days (~96 hours) of cultivation, cells were re-seeded and further cultivated until mid-log phase. All the experiments reported here were performed in cells subjected to this induction protocol and all times points indicated in the figures refer to the second round of induction. **C)** Western blotting analysis of whole cell extracts from *HUS1*^{Flox} cells collected at the indicated time points after RAP induction; HUS1 levels were assessed with anti-HUS1 antibody and EF1α was used as loading control. Despite the complete excision of *HUS1* from genome (see Figure S2A), the protein is still detectable after the first round of induction, likely due to its high stability. After the second round of induction HUS1 is not detectable.

Supplementary Figure 3

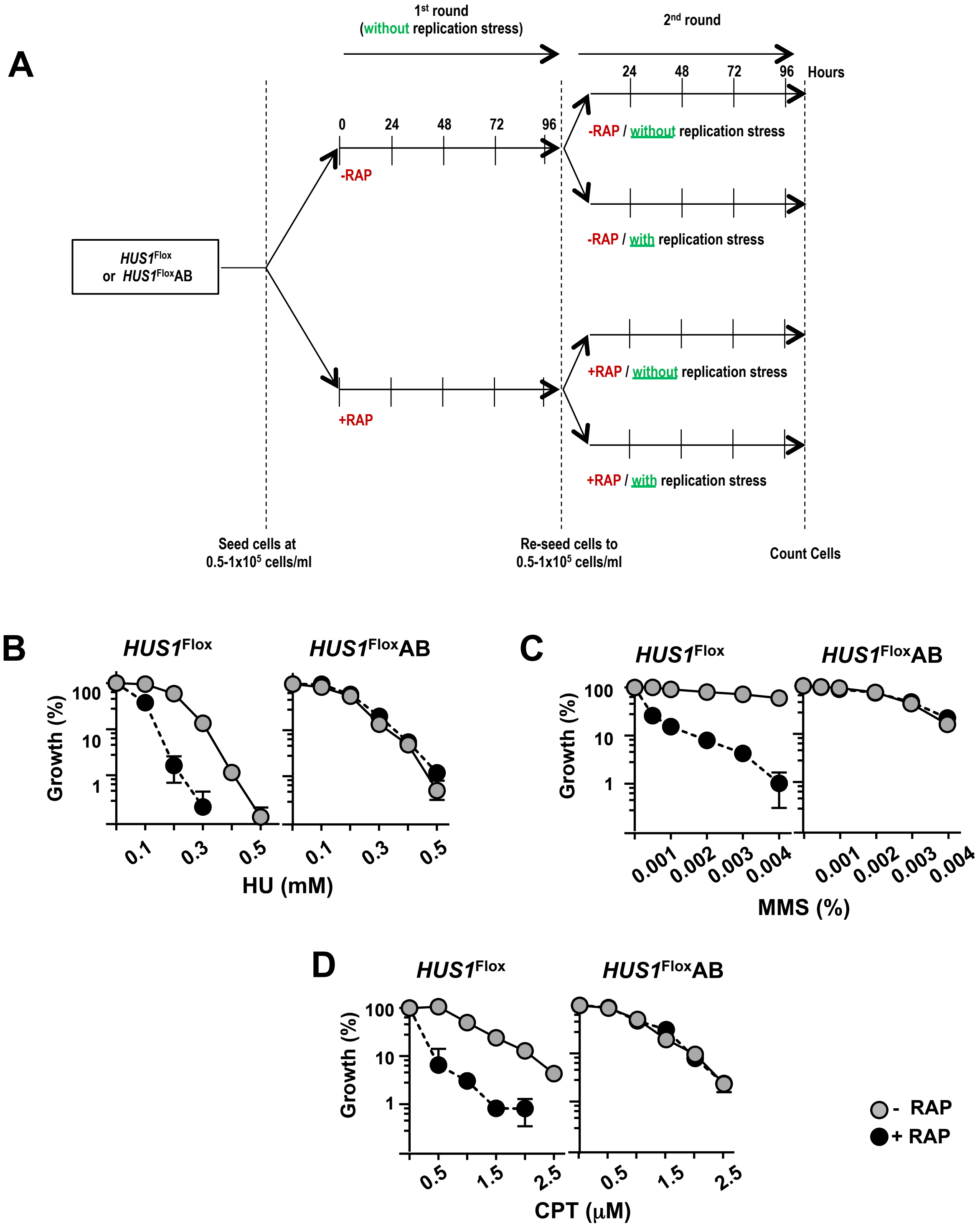

Figure S3. **Effect of *HUS1* KO in the resistance to replication stress-causing agents**. **A) I**llustration of the experimental design to evaluate resistance to replication stress-causing agents (B-D). Cells were seeded in medium with or without RAP, in the absence of any replication stress-causing agent. After the first round of induction (~96 hours), cells were re-seeded and further subjected to a second round of induction in medium with or without replication stress-causing agents at various concentration. After ~96 hours of the second round of induction, cell density in each condition was determined. **B – D)** Relative growth of cells incubated with the indicated concentration of hydroxyurea (HU), methyl methanesulfonate (MMS) or campthotecin (CPT) in the second round of induction; growth in each concentration is expressed as percentage of proliferation relative to cells cultivated without the genotoxic drugs; error bars depict SD.

Supplementary Figure 4

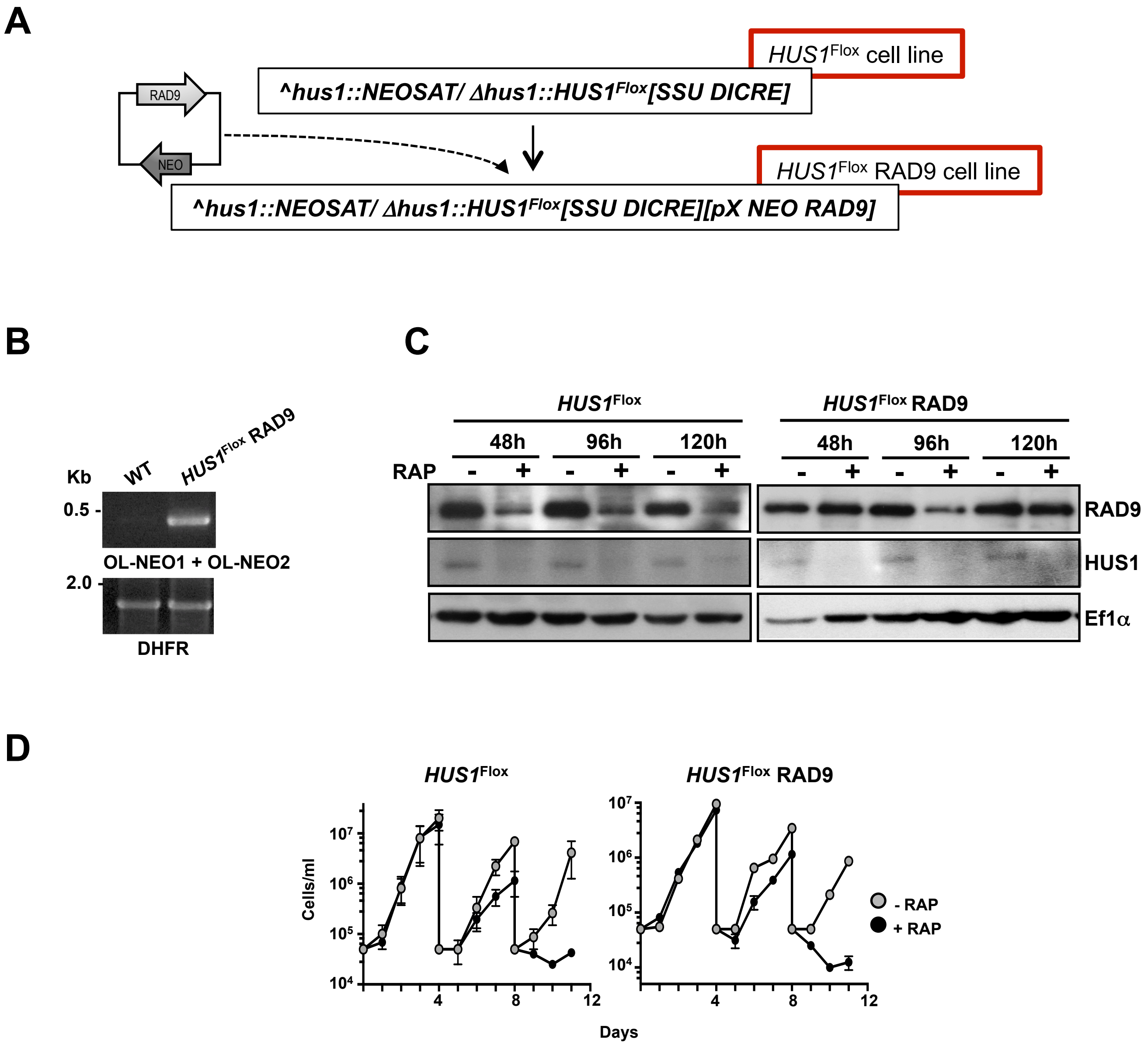

Figure S4. Characterization of the *HUS1*^{Flox}[pXG-RAD9] cell line. A) Schematic illustration of steps followed for the generation of the *HUS1*^{Flox}RAD9 cell line; the *HUS1*^{Flox} cell line (Figure S1) was transfected with a pXG1 NEO plasmid containing the RAD9 coding sequence resulting in the cell line that expresses RAD9 from an episome. **B)** Genomic DNA from the indicated cell lines was PCR amplified using the indicated set of primers; PCR products was resolved in agarose gels and visualized with Ethidium Bromide; OL-NEO1 and OL-NEO2 were used to demonstrate the presence of pXG-RAD9 plasmid in the *HUS1*^{Flox}[pXG-RAD9] cell line; DHFR was used as control. **C)** Western blotting analysis of whole cell extracts from *HUS1*^{Flox} and *HUS1*^{Flox}[pXG-RAD9] cells collected at the indicated time points of the second round of RAP induction; HUS1 and RAD9 levels were assessed with specific antibodies and EF1α was used as loading control. **D)** Representative growth curves of *HUS1*^{Flox} and *HUS1*^{Flox}[pXG-RAD9] cell lines in the presence or absence of RAP; cells were seeded at ~ 5x10^4 cells/ml in day 0 and re-seeded every 4 days (96 hours) to complete three rounds of induction (days 0-4: first round; days 4-8: second round; third round was seeded from day 8 cultures); cell density was assessed every 24 hours and error bars depict SEM; red arrowheads indicate the points analysed in the western blot shown in (D) and correspond to 48, 96 and 120 hours of the second round.

Supplementary Figure 5

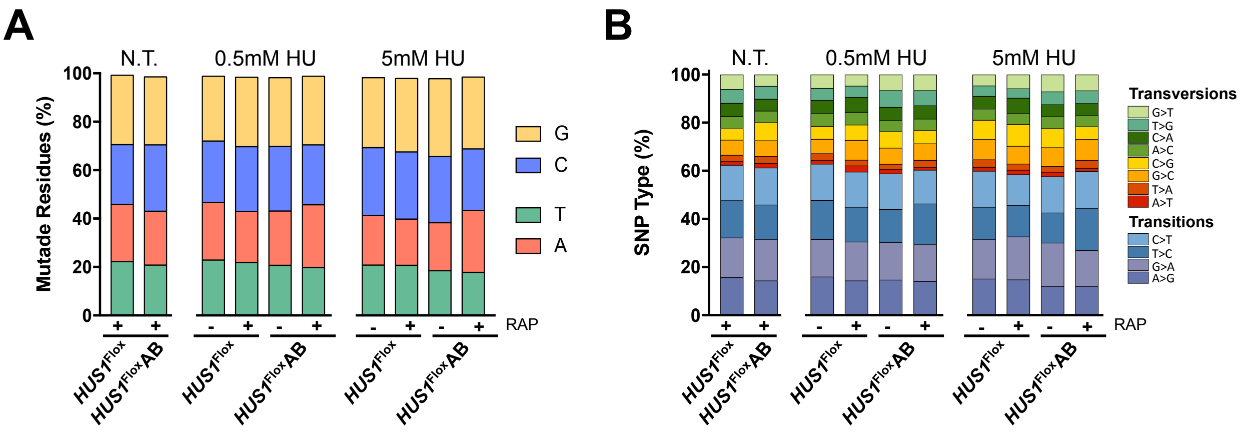

Figure S5. Quantification of SNP mutation types. A) Proportion of each type of residues that was different from the reference genome. **B)** Proportion of transitions and transversions SNPs events in each indicated condition.

Supplementary Figure 6

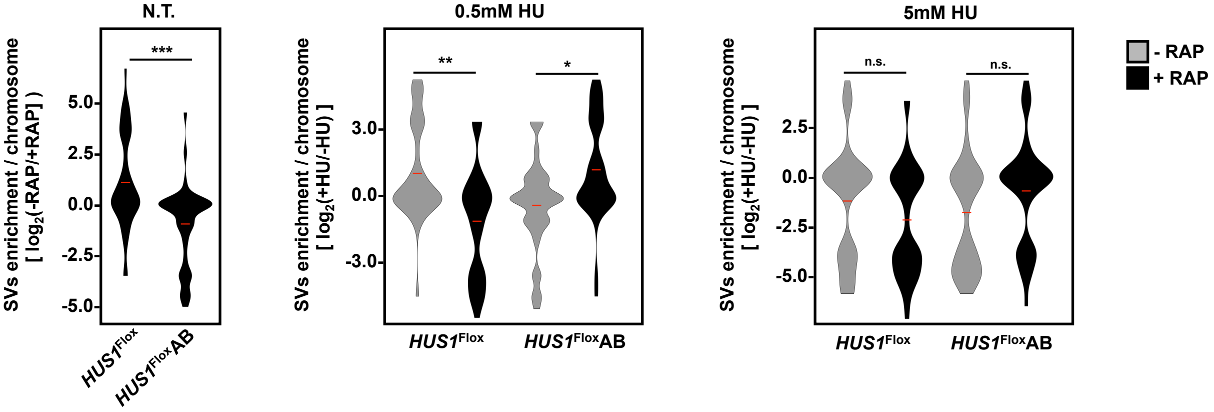

Figure S6. Analysis of Structural Variants (SVs). SVs (Deletions, Duplications, Translocations and Insertions) were identified using DELLY software (Rausch et al., (2012) *Bioinformatics*, **28**, i333-i339). The data is represented as log2 of the ration of the number of new SVs identified per chromosome after RAP induction and/or HU treatments. The data are represented as violin plots, where shape indicates the distribution of pooled data and horizontal red lines indicate mean; differences were tested with Kruskal-Wallis test and are as indicated: (***), p=0.0028; (**), p=0.0044; (*), p=0.0402; n.s., not significant.

Supplementary Figure 7

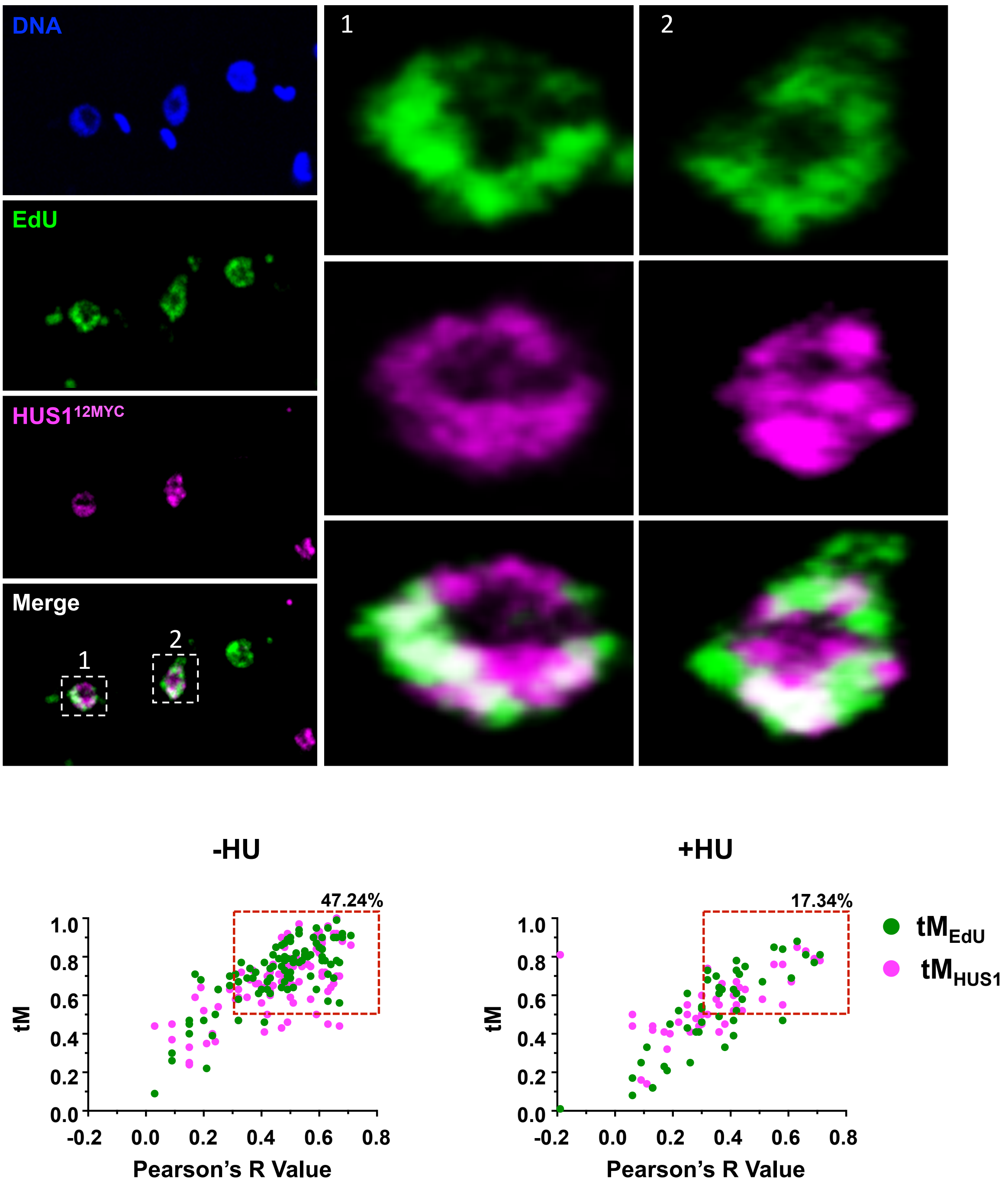

Figure S7. Quantitative analysis of co-localization between endogenously tagged HUS1 and EdU. Cells expressing HUS1-12xMYC from the endogenous locus were left untreated (-HU) or treated with 5 mM HU (+HU) for 5 hours. HU was removed and cells were incubated with 10mM EdU, fixed and processed for sequential EdU and HUS1-12xMYC detection. Images corresponding to a single confocal Z-slice were deconvolved using BlindDblur (LAS AF Leica Software). Co-localization between HUS1-12xMYC and EdU in the nucleus was quantified in single confocal slices using Coloc 2 Fiji plugin. Two factors were took into account to determine relevant co-localization events: Pearson's R value (spacial correlation between the two signals) and Thresholded Mander's (tM_{EdU}, proportion of EdU signal overlapping with HUS1-12xMYC; tM_{HUS1}, proportion of HUS1-12xMYC signal overlapping with EdU). Red box indicate nucleus where co-localization where above 0.5 for all parameters.

Supplementary Figure 8

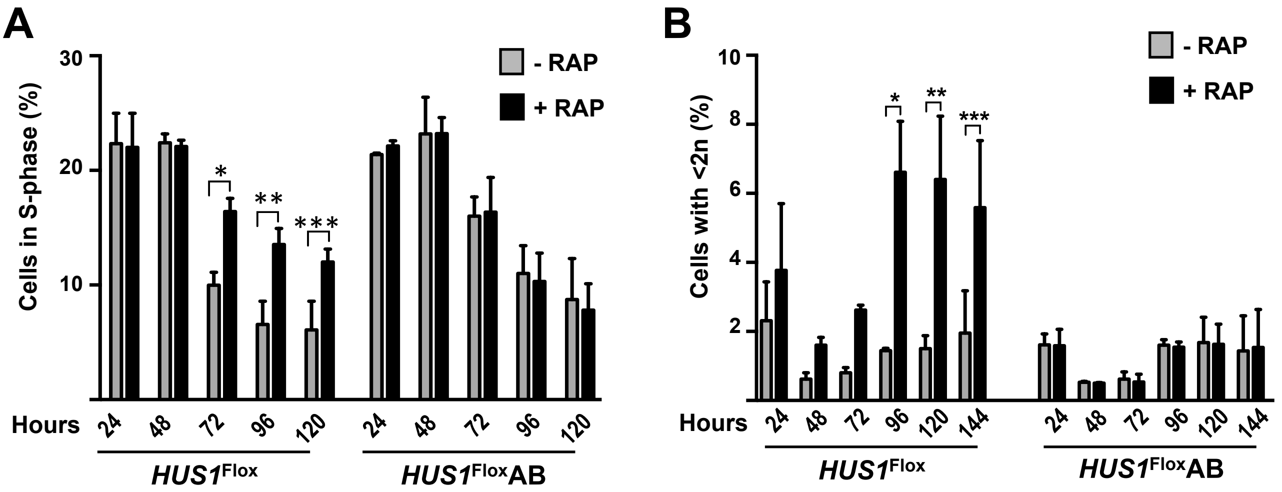

Figure S8. **Analysis of DNA content profile through time course of *HUS1* KO induction**. Quantitative evaluation from three independent experiments of the proportion of cells in S-phase (**A)** and cells with <2n DNA content **(B)** as determined by PI staining followed by flow cytometry analysis. A representative histogram used for these quantifications is shown in Fig. 2C, in the main text. Differences were tested with one way ANOVA-test. In A: (*), p =0.0271; (**), p =0.0064; (***), p =0.0406. In B: (*), p =0.0003; (**), p =0.0005, (***), p =0.0257. Error bars indicate SEM.

Supplementary Figure 9

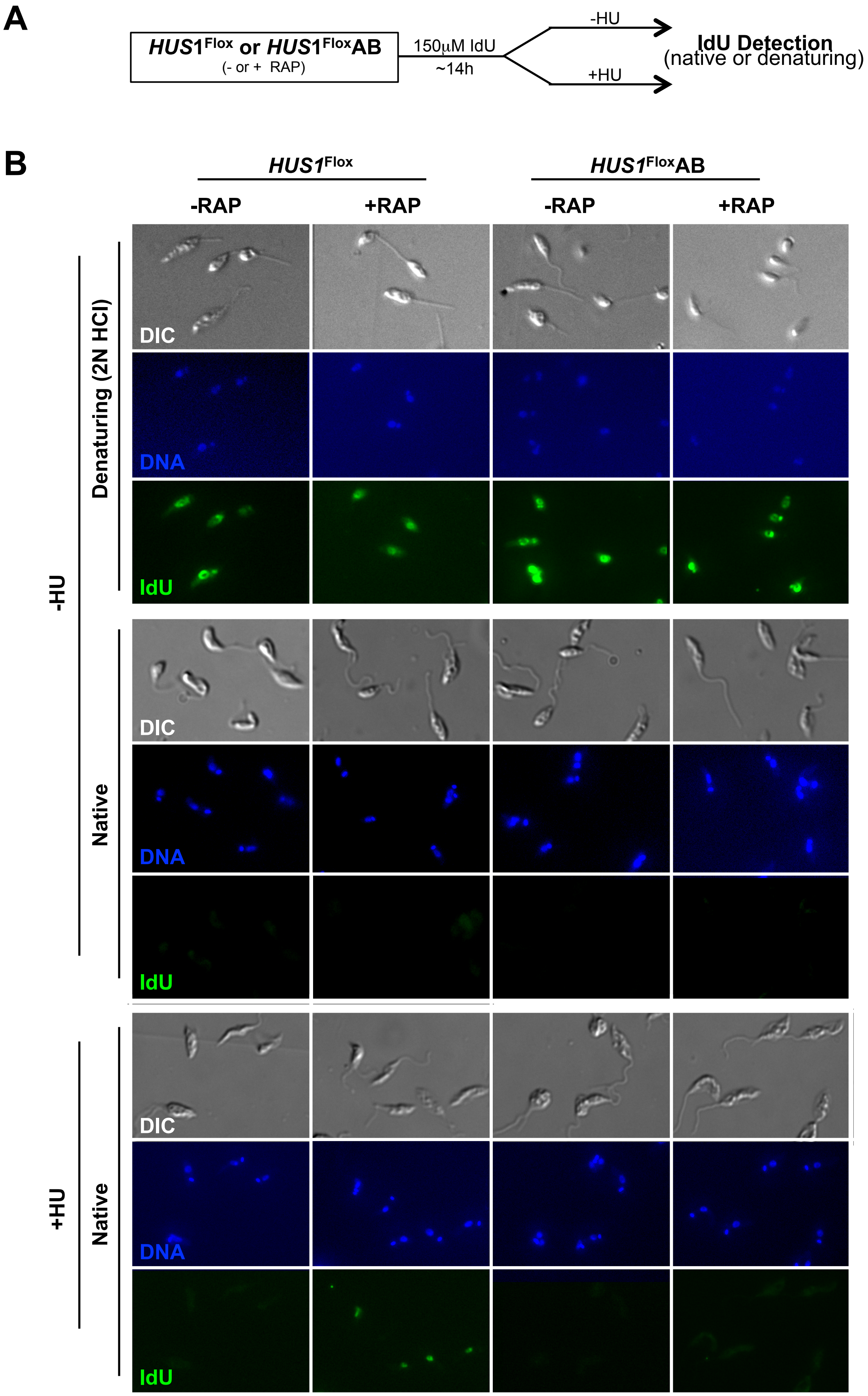

Supplementary Figure 9

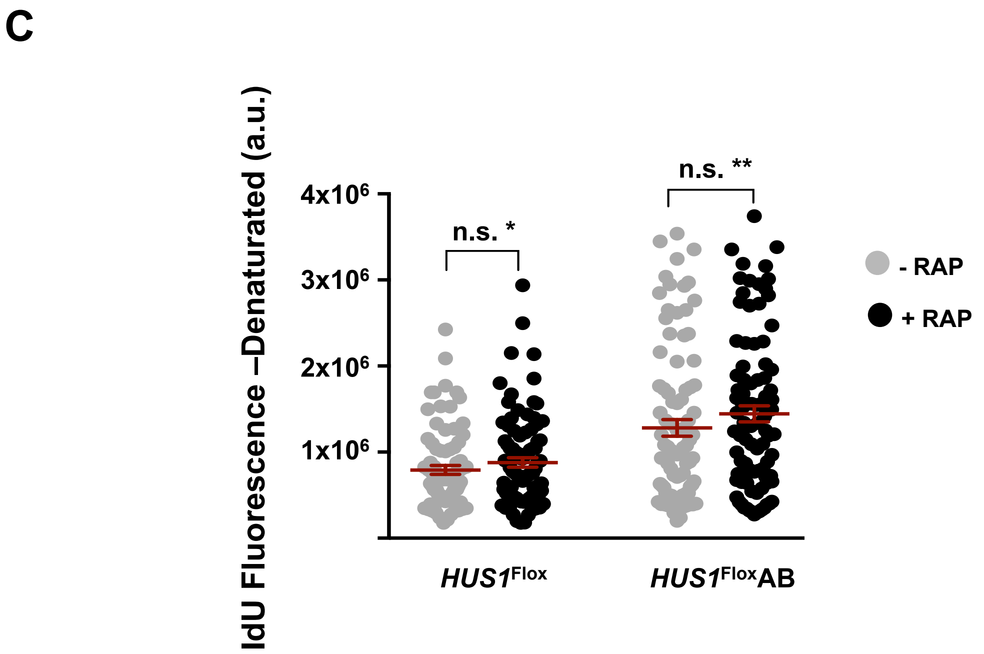

Figure S9. **Protocol for ssDNA detection accumulation using metabolic labelling of DNA with IdU**. **A)** Schematic illustration of ssDNA detection approach; all cell lines were incubated ~14 hours with IdU and then exposed to 5mM HU (~8 hours) and processed to IdU detection. **B)** Immunofluorescence analysis of IdU under native or denaturing conditions, as indicated. Note that, under denaturing conditions, all cells are IdU positive, whereas no detectable signal is observed under native detection conditions. On the other hand, HUS1 KO cells, but not control cells, present substantial IdU signal after HU treatment under native conditions of detection. **C)** Quantification of IdU fluorescence detected under denaturing conditions. Signal from individual cells was determined with ImageJ software and plotted as shown; a.u., arbitrary units; no significant (n.s.) difference in IdU incorporation was observed between the conditions of interest, as determined by Kruskal-Wallis test. Thus, differences in IdU signal detected under native conditions can not be attributed to an intrinsic difference in overall incorporation of IdU. (*), p=0.3446. (**), p=0.1125

Supplementary Figure 10

A

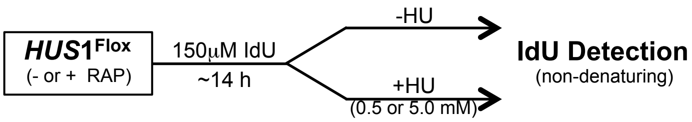

B

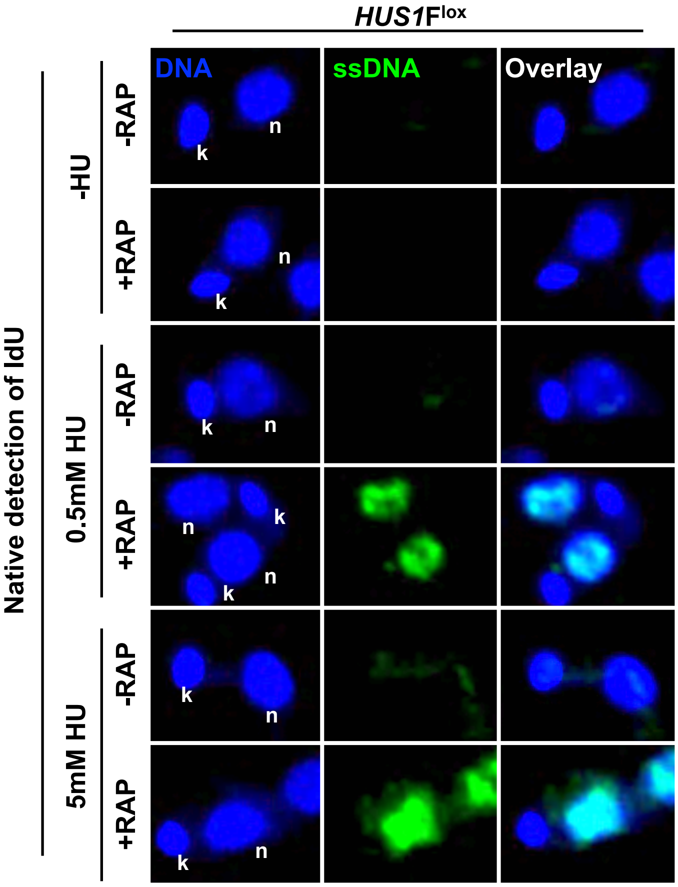

Figure S10. **Analysis of ssDNA accumulation upon in *HUS1* KO cells upon chronic and acute replication stress**. **A)** Schematic illustration of ssDNA detection approach. **B)** Immunofluorescence analysis of ssDNA upon mild and acute replication stress. Images were acquired with a multiphoton microscope (Zeiss).

Supplementary Figure 11

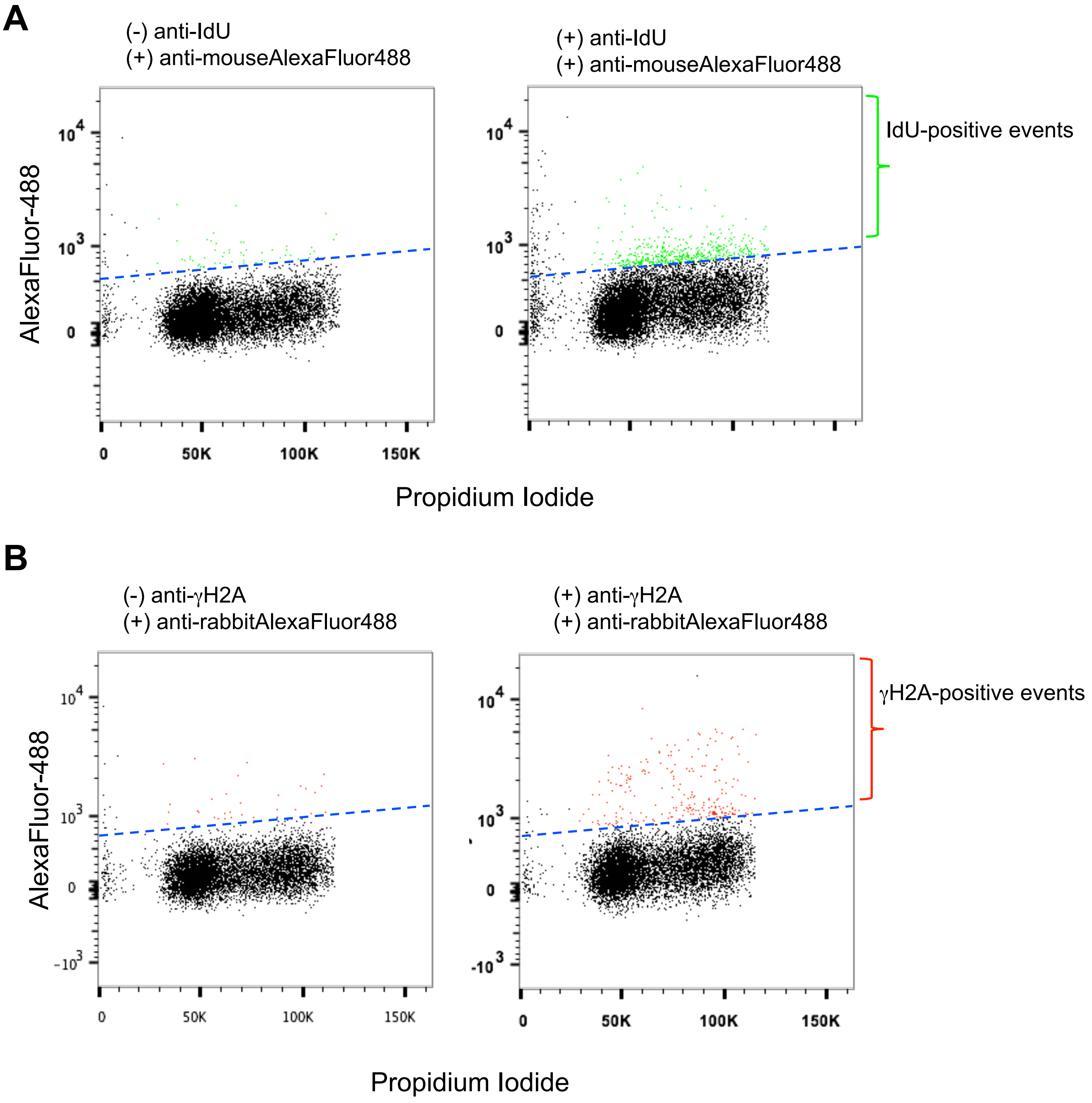

Figure S11. **Gate strategy for IdU and γH2A detection by FACS**. Representative dot plot from flow cytometry analysis for detection of ssDNA **(A)** and γH2A **(B)**; blue dotted lines indicate threshold to discriminate positive from negative events; the discrimination was based on signal differences when comparing cells incubated or not with primary antibody.

Supplementary Figure 12

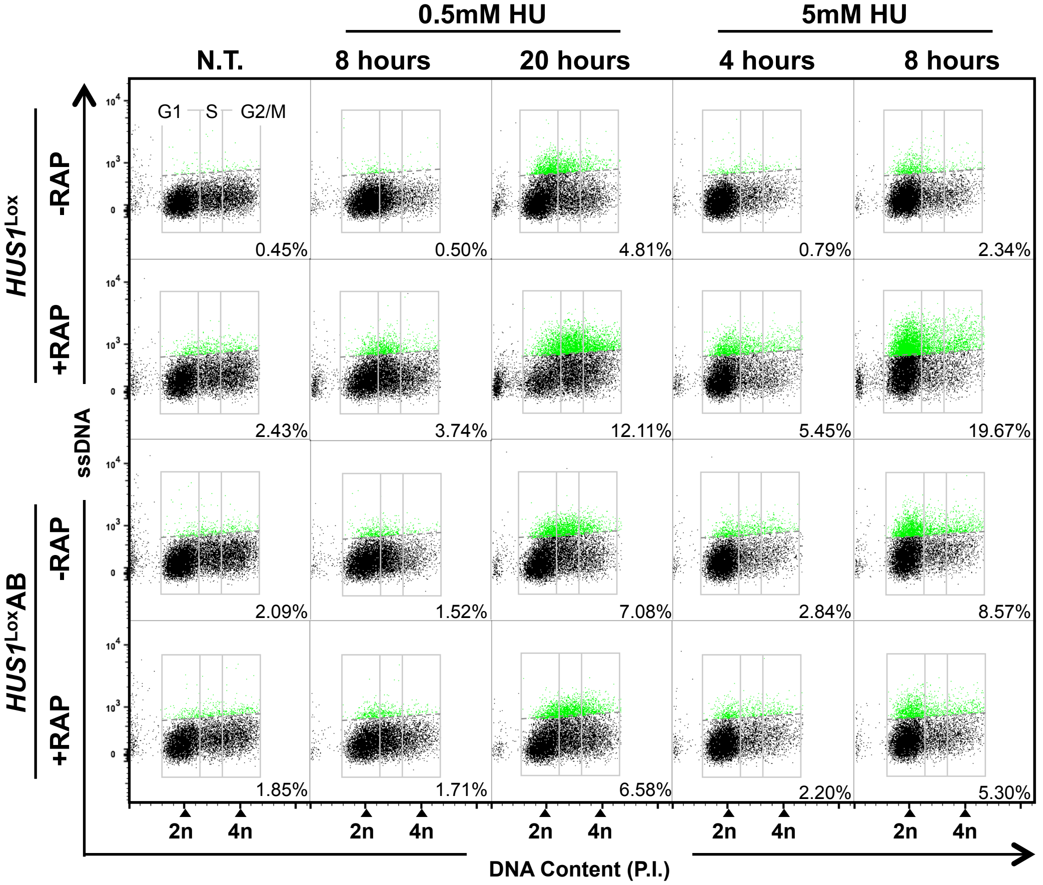

Figure S12. **Analysis of ssDNA accumulation using flow cytometry**. The same experiment shown in Fig. 2F; in here, the profile of *HUS1*^{Flox}AB cells subjected to the same conditions were included. After 48 hours of induction cells were incubated with 150μM IdU for ~14 hours; then, cells were left untreated (N.T.) or treated with the indicated HU concentration for the indicated period of time and then processed for IdU detection under native conditions.

Supplementary Figure 13

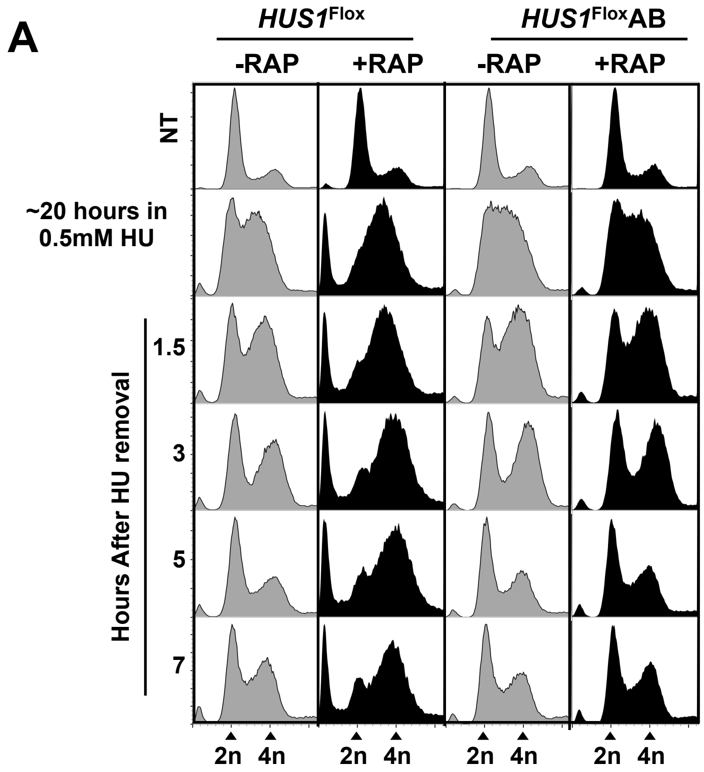

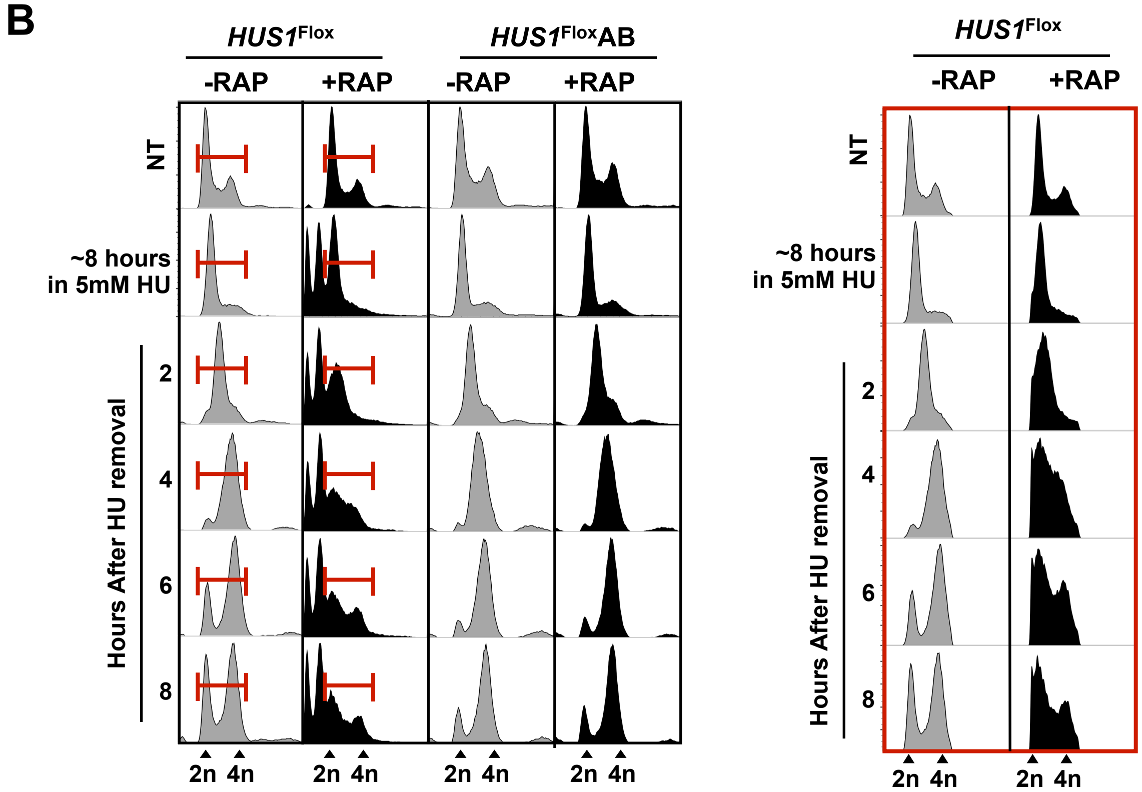

Figure S13. **Cell cycle progression analysis after replication stress**. **A)** and **B)** At 48 hours after induction, cells were left untreated (N.T.) or treated ~20 hours with 0.5mM HU (A) or ~8 hours with 5mM HU (B) and then re-seeded in HU-free medium; cells were collected at the indicated time points after HU removal, fixed, stained with Propidium Iodide and analyzed by FACS. In (B), gates represented as red lines in panels at left were used to generate histograms in panels at right. With, the visual perception of faster progression of HUS1 KO cells is facilitates. Note, for instance, that uninduced cells seems to start transition between G2/M back to G1 at 6 – 8 hours after HU removal. In contrast, HUS1 KO cells seems to be already cycling by this time point.

Supplementary Figure 14

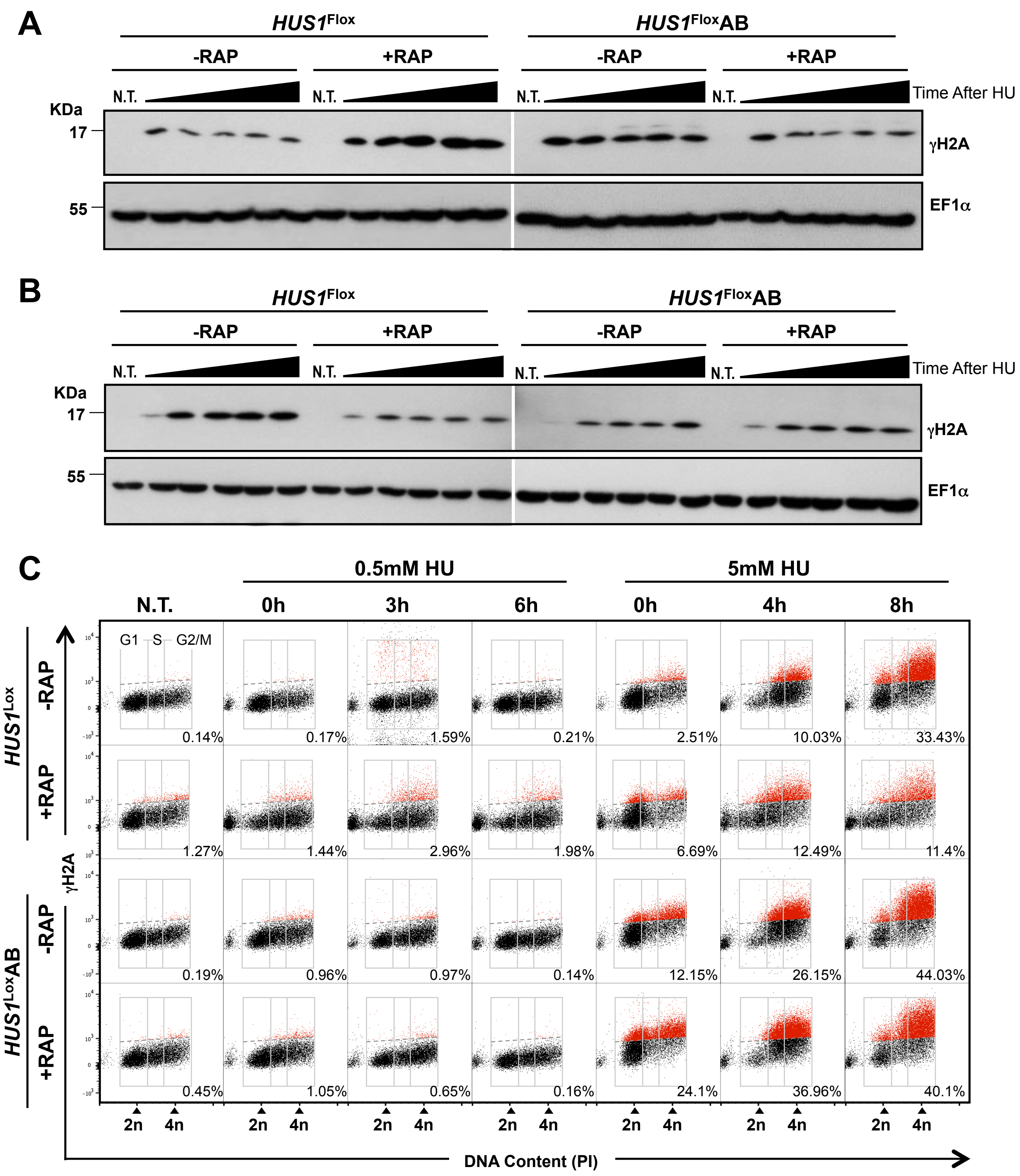

Figure S14. **Analysis of γH2A levels after replication stress removal**. **A)** and **B)** Western blotting analysis of whole cell extracts from *HUS1*^{Flox} and *HUS1*^{Flox}AB cells; at 48 hours after induction, cells were left untreated (N.T.) or treated ~20 hours with 0.5mM HU (A) or ~8 hours with 5mM (B) HU and then re-seeded in HU-free medium; cells were collected at 0, 1.5, 3, 5 and 7 hours (A) or 0, 2, 4, 6 and 8 (B) hours after HU removal; extracts were probed for γH2A levels and EF1α was used as loading control. **C)** The same experiment shown in Fig. 4C, in which here the profile of *HUS1*^{Flox}AB cells subjected to the same conditions were included. At 48 hours after induction, cells were left untreated (N.T.) or treated ~20 hours with 0.5mM HU or ~8 hours with 5mM HU and then re-seeded in HU-free medium; cells were collected at the indicated time points after HU removal, fixed and subjected to γH2A detection.

Supplementary Table 1

Primer Name	Orientation	Sequence (5' – 3')
OL2380	Forward	CATTCCGTGCGAAAGCCGG
OL4102	Reverse	GATGGTTTCCACCTGCAC
OL-5UTR(HUS1)	Forward	TCCACGTTCCTTTGTACGTG
OL-SAT	Reverse	TTGGGGCCCTGTGGACCCTTGGCGGC
OL-HUS1$_{Fw}$	Forward	ATGCGCTTCAAGGCGACCC
OL-HUS1$_{Rv}$	Reverse	CGCCCTTGCGGGGATATAGG
OL-15	Reverse	CCGTGGGCTTGTACTCGGTCA
OL-NEO1	Forward	TGTTGGGTCGTTTGTTCGGA
OL-NEO2	Reverse	CGAGGAGATCGAGGGAAGGA
G6PDH1	Forward	ACCGCATTGACCACTACCTC
G6PDH2	Reverse	GATGTTGTTCGAGTTCCAC
